# Supplementary material for: Using Forum Theater as a Teaching Tool to Combat Patient Bias Directed Toward Health Care Professionals
Source: MedEdPORTAL. 2020 Nov 20;16:11022. doi: 10.15766/mep_2374-8265.11022 (PMC7678028; doi:10.15766/mep_2374-8265.11022)
Supplement: Supplementary file 1 — Presentation.pptxFacilitator Guide.docxPrefilmed Scenario.m4vEvaluation Form.docx [file mep_2374-8265.11022-s001.zip › A. Presentation.pptx]

## Slide 1
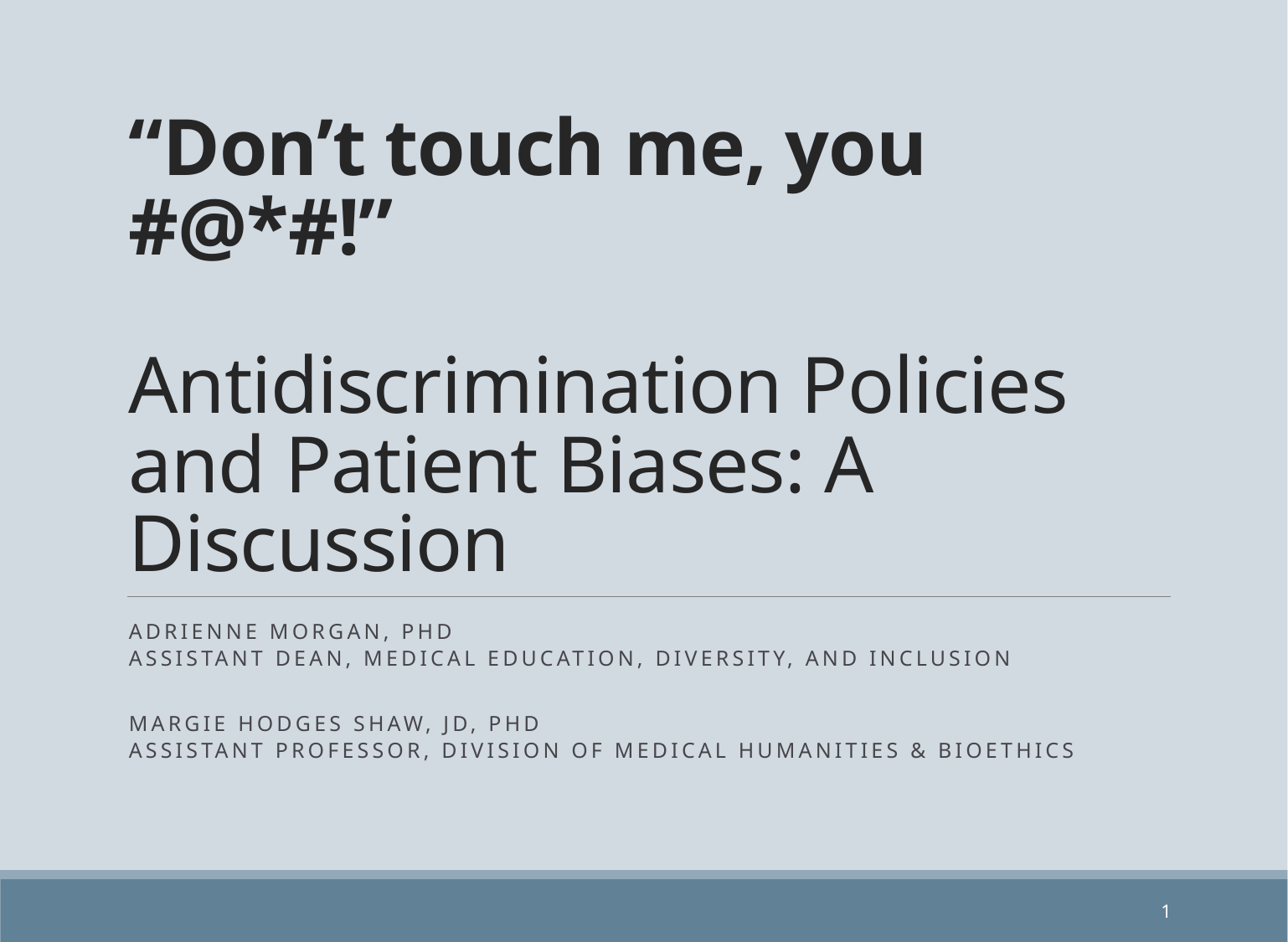

# “Don’t touch me, you #@*#!” Antidiscrimination Policies and Patient Biases: A Discussion
Adrienne Morgan, PhD
Assistant Dean, Medical Education, Diversity, and Inclusion
Margie Hodges Shaw, JD, PhD
Assistant Professor, Division of Medical Humanities & Bioethics
1

## Slide 2
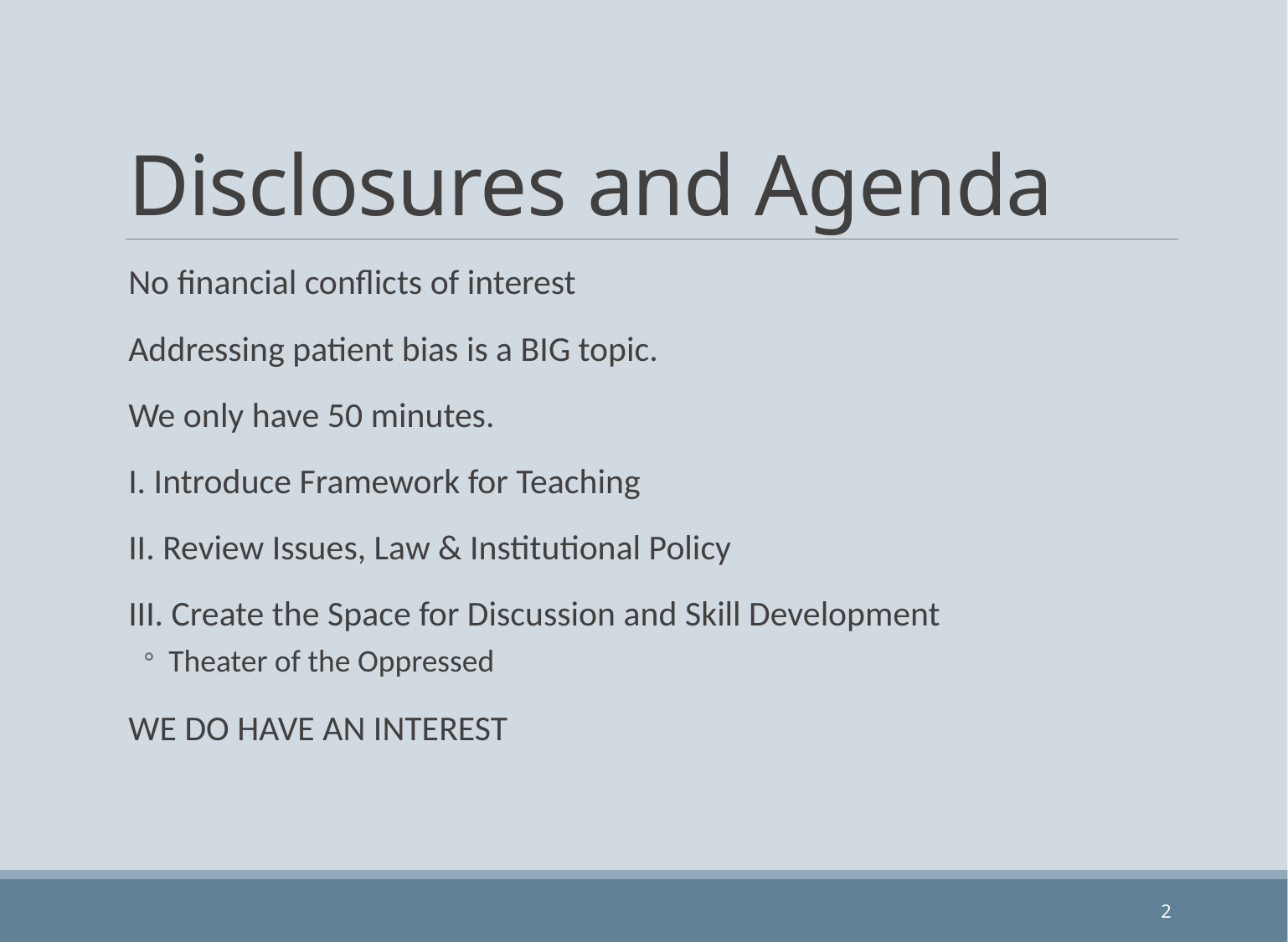

# Disclosures and Agenda
No financial conflicts of interest
Addressing patient bias is a BIG topic.
We only have 50 minutes.
I. Introduce Framework for Teaching
II. Review Issues, Law & Institutional Policy
III. Create the Space for Discussion and Skill Development
Theater of the Oppressed
WE DO HAVE AN INTEREST
2

## Slide 3
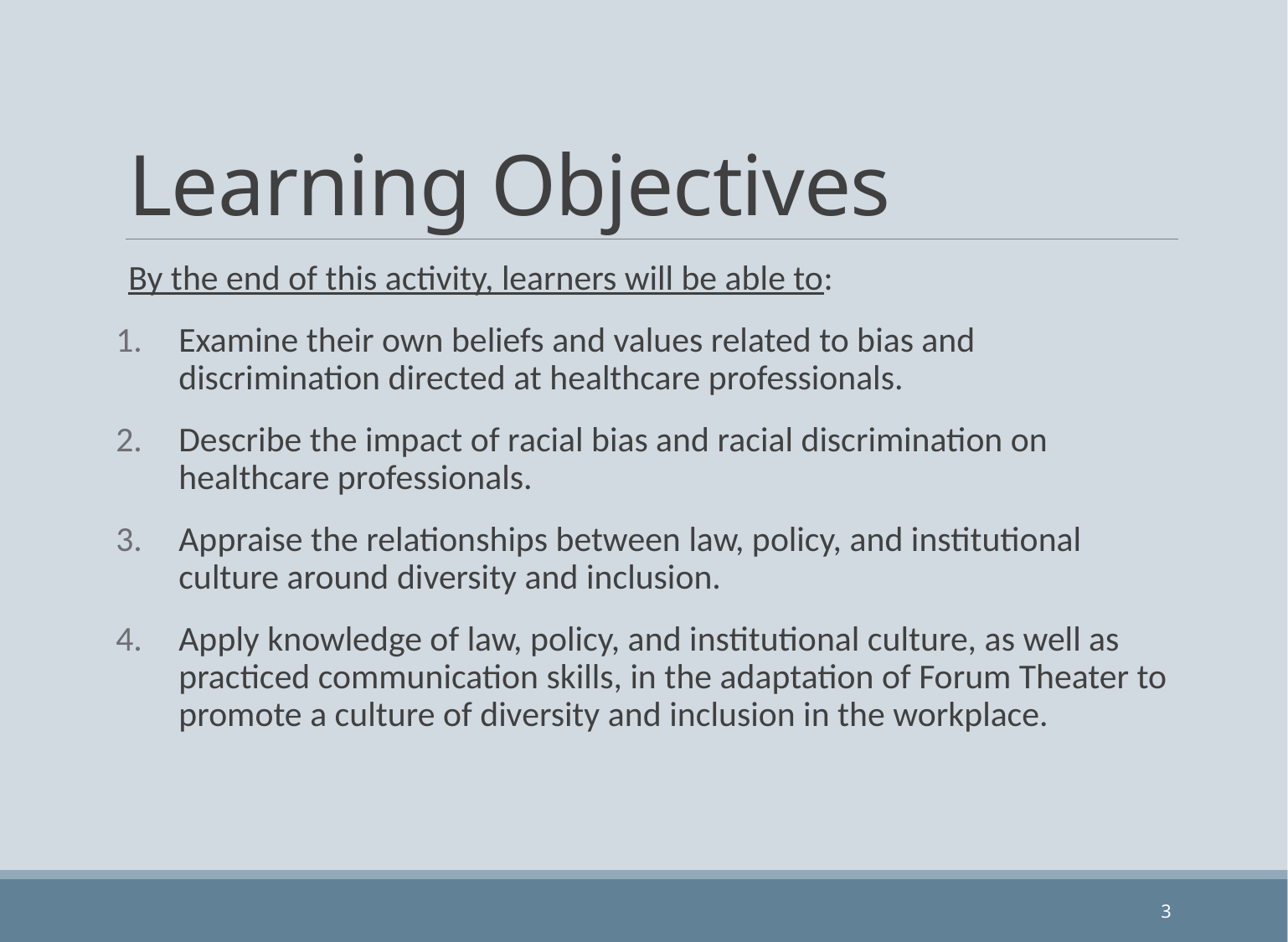

# Learning Objectives
By the end of this activity, learners will be able to:
Examine their own beliefs and values related to bias and discrimination directed at healthcare professionals.
Describe the impact of racial bias and racial discrimination on healthcare professionals.
Appraise the relationships between law, policy, and institutional culture around diversity and inclusion.
Apply knowledge of law, policy, and institutional culture, as well as practiced communication skills, in the adaptation of Forum Theater to promote a culture of diversity and inclusion in the workplace.
3

## Slide 4
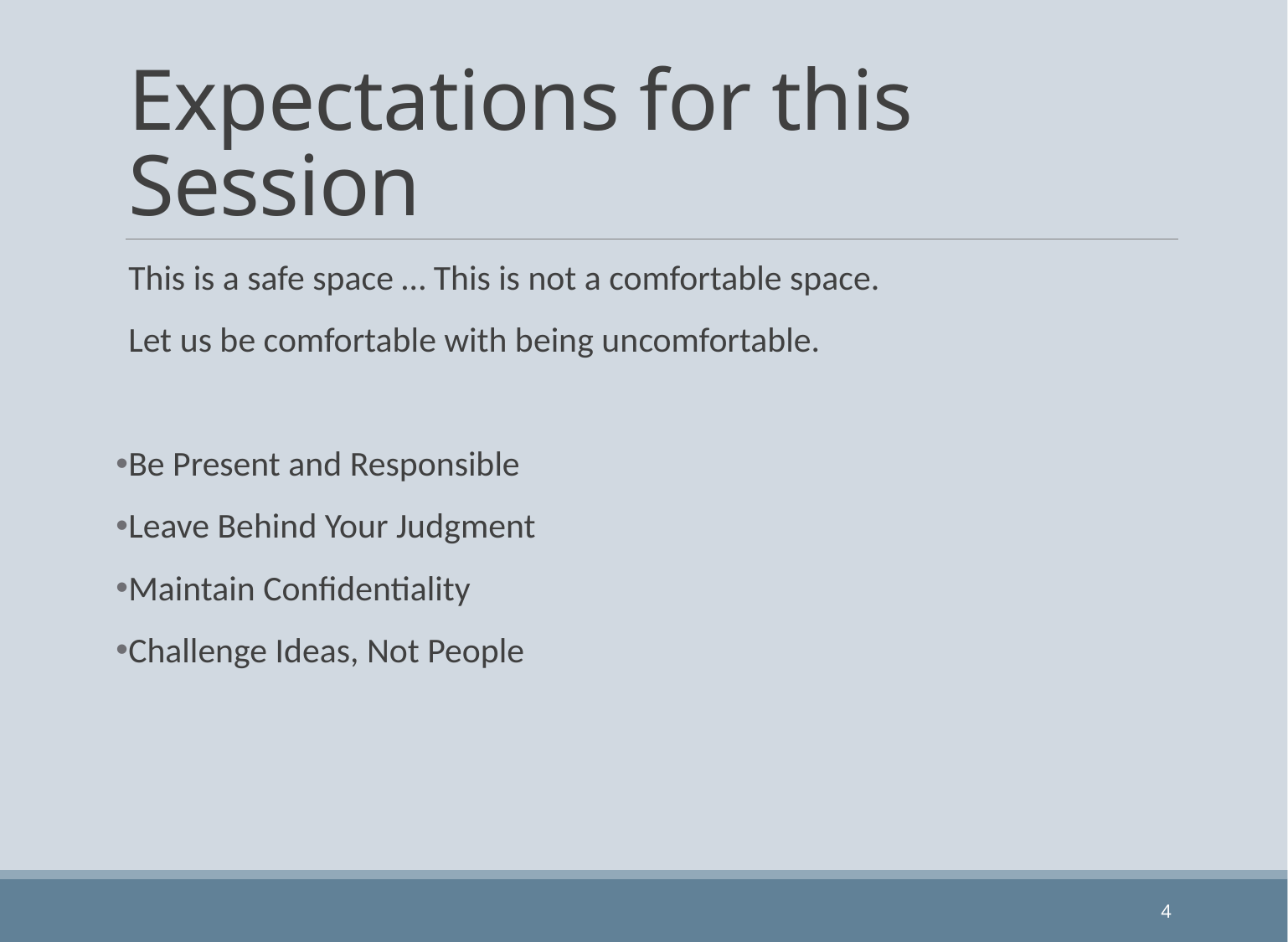

# Expectations for this Session
This is a safe space … This is not a comfortable space.
Let us be comfortable with being uncomfortable.
Be Present and Responsible
Leave Behind Your Judgment
Maintain Confidentiality
Challenge Ideas, Not People
4

## Slide 5
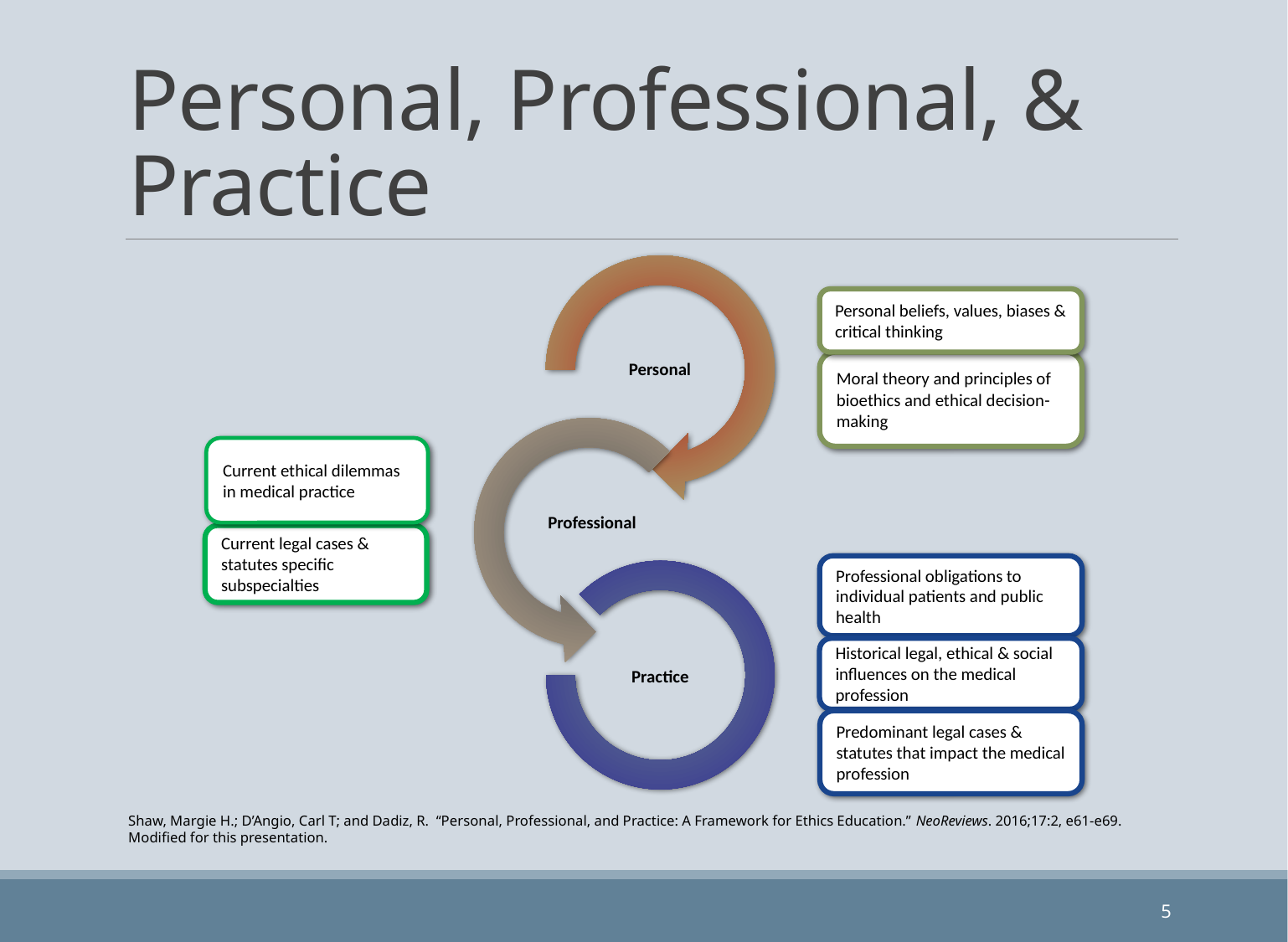

# Personal, Professional, & Practice
Personal beliefs, values, biases & critical thinking
Moral theory and principles of bioethics and ethical decision-making
Current ethical dilemmas in medical practice
Current legal cases & statutes specific subspecialties
Professional obligations to individual patients and public health
Historical legal, ethical & social influences on the medical profession
Predominant legal cases & statutes that impact the medical profession
Shaw, Margie H.; D’Angio, Carl T; and Dadiz, R. “Personal, Professional, and Practice: A Framework for Ethics Education.” NeoReviews. 2016;17:2, e61-e69. Modified for this presentation.
5

## Slide 6
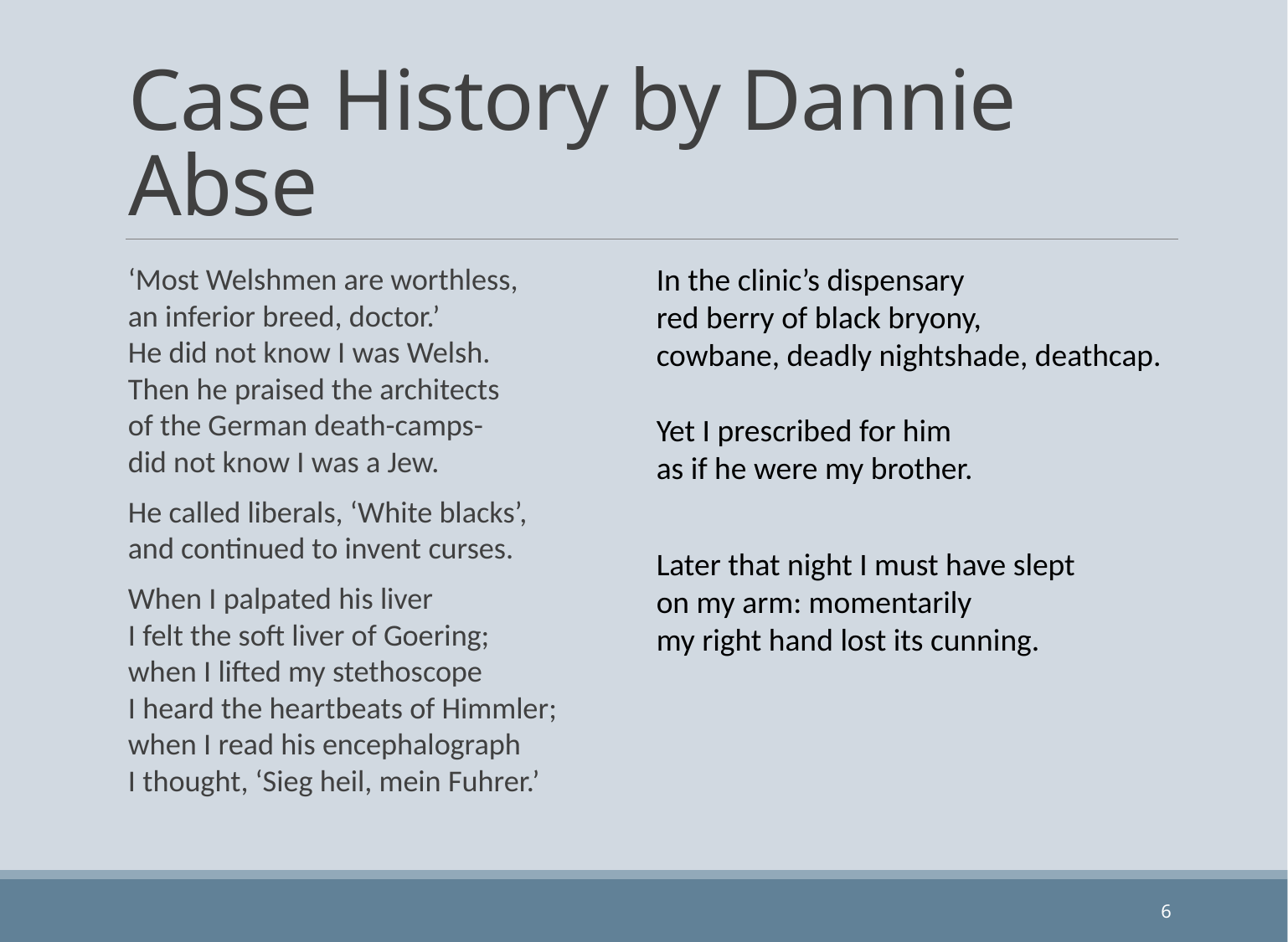

# Case History by Dannie Abse
‘Most Welshmen are worthless,
an inferior breed, doctor.’
He did not know I was Welsh.
Then he praised the architects
of the German death-camps-
did not know I was a Jew.
He called liberals, ‘White blacks’,
and continued to invent curses.
When I palpated his liver
I felt the soft liver of Goering;
when I lifted my stethoscope
I heard the heartbeats of Himmler;
when I read his encephalograph
I thought, ‘Sieg heil, mein Fuhrer.’
In the clinic’s dispensary
red berry of black bryony,
cowbane, deadly nightshade, deathcap.
Yet I prescribed for him
as if he were my brother.
Later that night I must have slept
on my arm: momentarily
my right hand lost its cunning.
6

## Slide 7
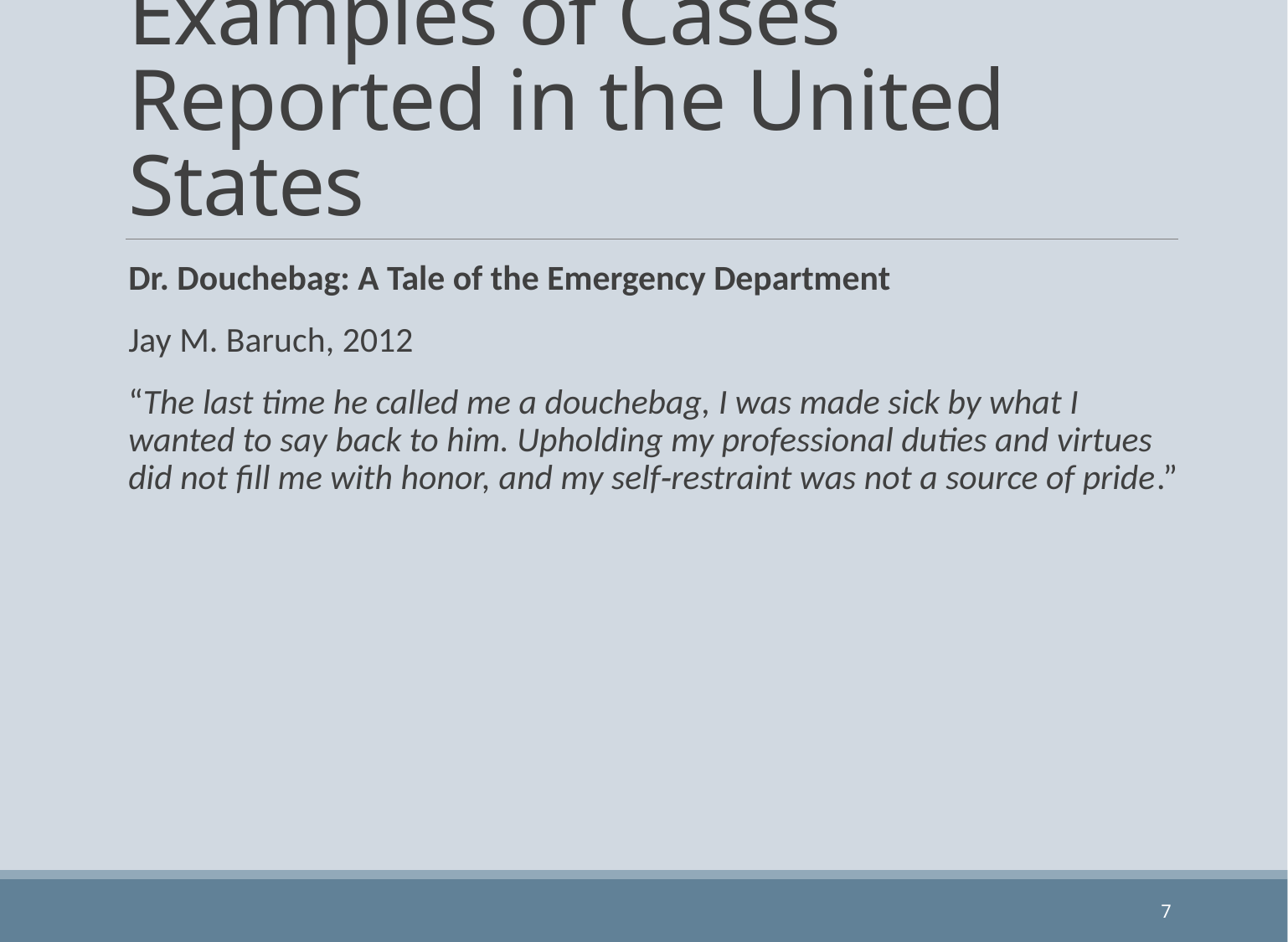

# Examples of Cases Reported in the United States
Dr. Douchebag: A Tale of the Emergency Department
Jay M. Baruch, 2012
“The last time he called me a douchebag, I was made sick by what I wanted to say back to him. Upholding my professional duties and virtues did not fill me with honor, and my self‐restraint was not a source of pride.”
7

## Slide 8
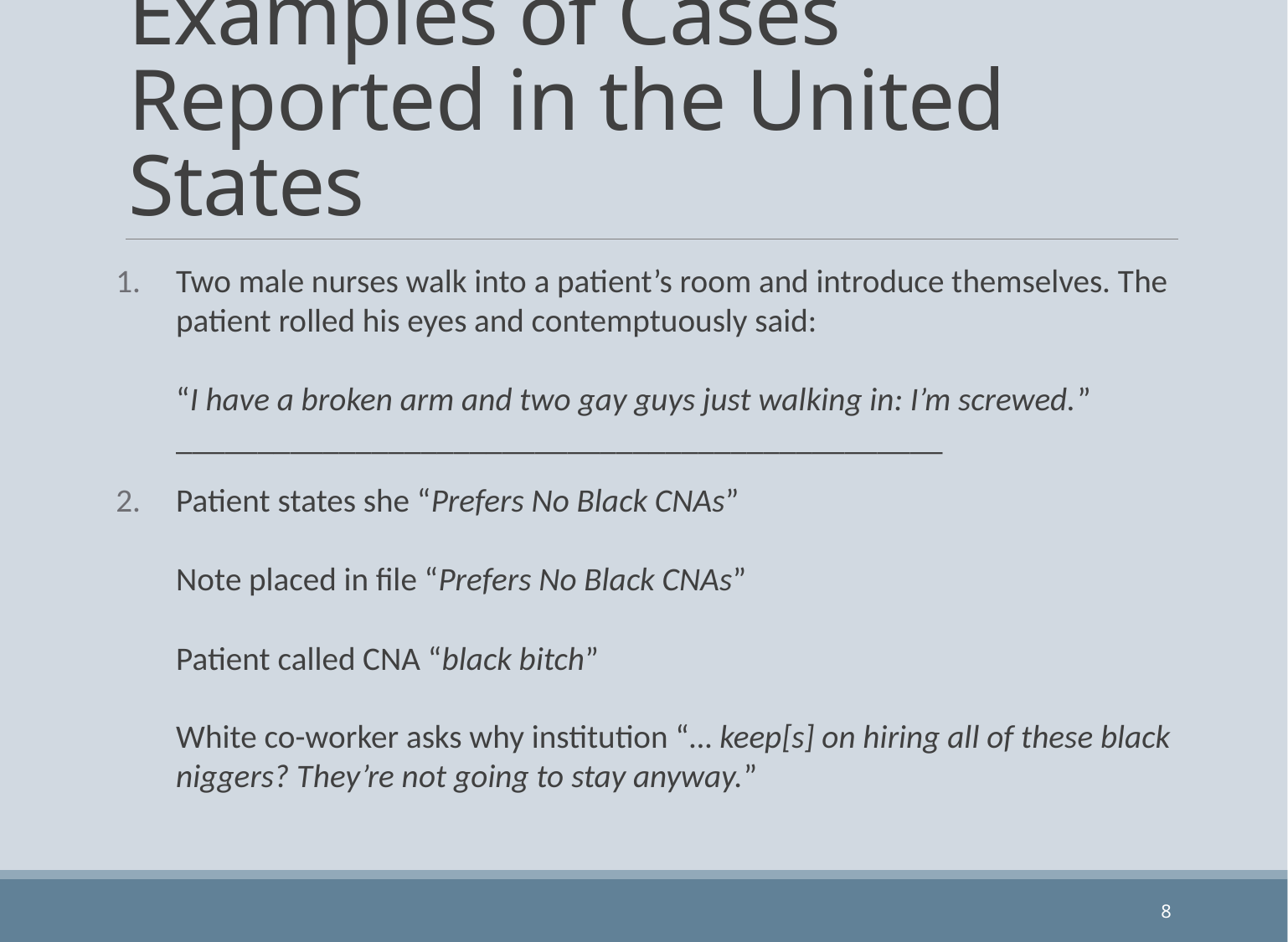

# Examples of Cases Reported in the United States
Two male nurses walk into a patient’s room and introduce themselves. The patient rolled his eyes and contemptuously said:
“I have a broken arm and two gay guys just walking in: I’m screwed.”
_______________________________________________
Patient states she “Prefers No Black CNAs”
Note placed in file “Prefers No Black CNAs”
Patient called CNA “black bitch”
White co-worker asks why institution “… keep[s] on hiring all of these black niggers? They’re not going to stay anyway.”
8

## Slide 9
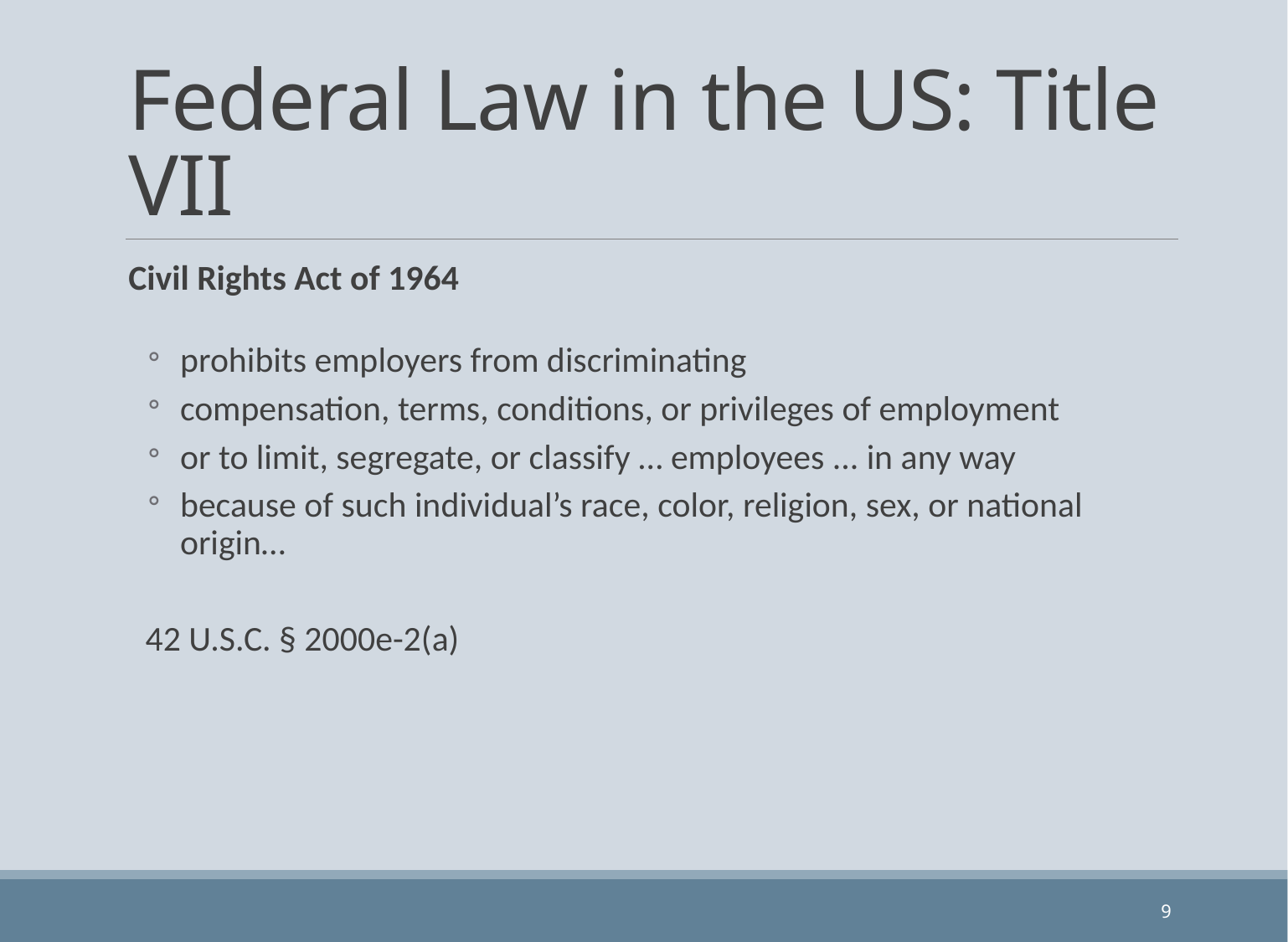

# Federal Law in the US: Title VII
Civil Rights Act of 1964
prohibits employers from discriminating
compensation, terms, conditions, or privileges of employment
or to limit, segregate, or classify … employees ... in any way
because of such individual’s race, color, religion, sex, or national origin…
42 U.S.C. § 2000e-2(a)
9

## Slide 10
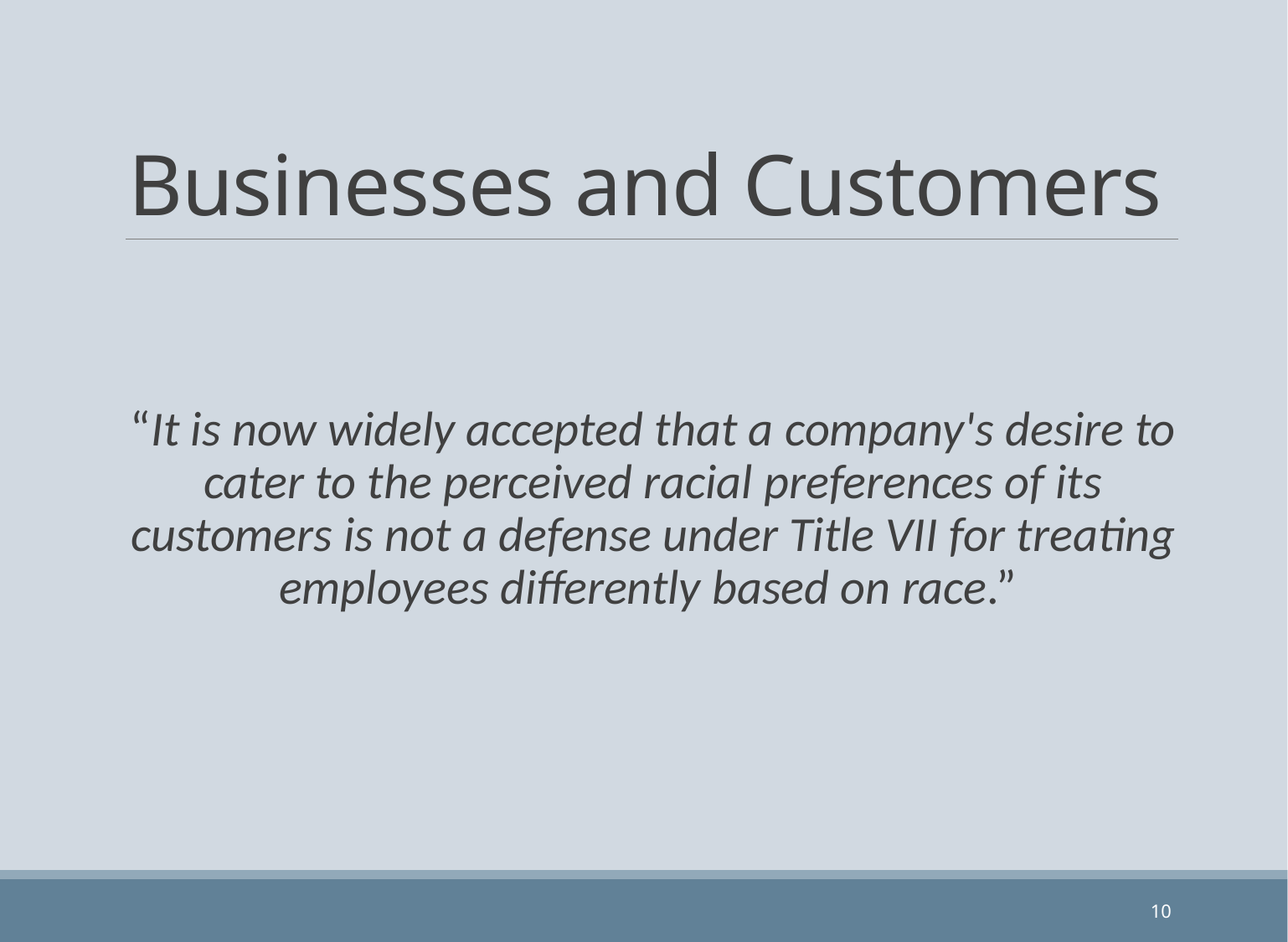

# Businesses and Customers
“It is now widely accepted that a company's desire to cater to the perceived racial preferences of its customers is not a defense under Title VII for treating employees differently based on race.”
10

## Slide 11
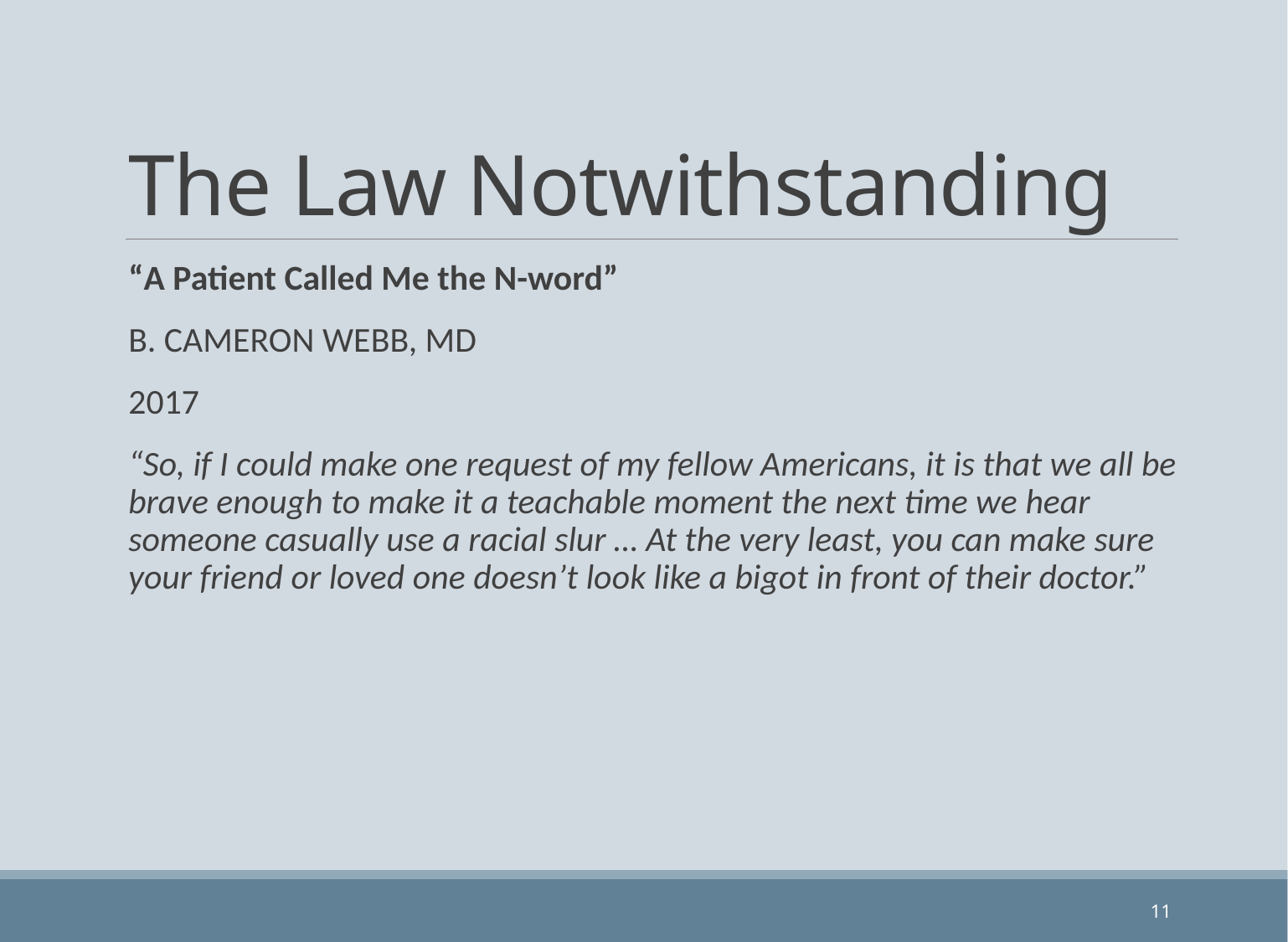

# The Law Notwithstanding
“A Patient Called Me the N-word”
B. CAMERON WEBB, MD
2017
“So, if I could make one request of my fellow Americans, it is that we all be brave enough to make it a teachable moment the next time we hear someone casually use a racial slur … At the very least, you can make sure your friend or loved one doesn’t look like a bigot in front of their doctor.”
11

## Slide 12
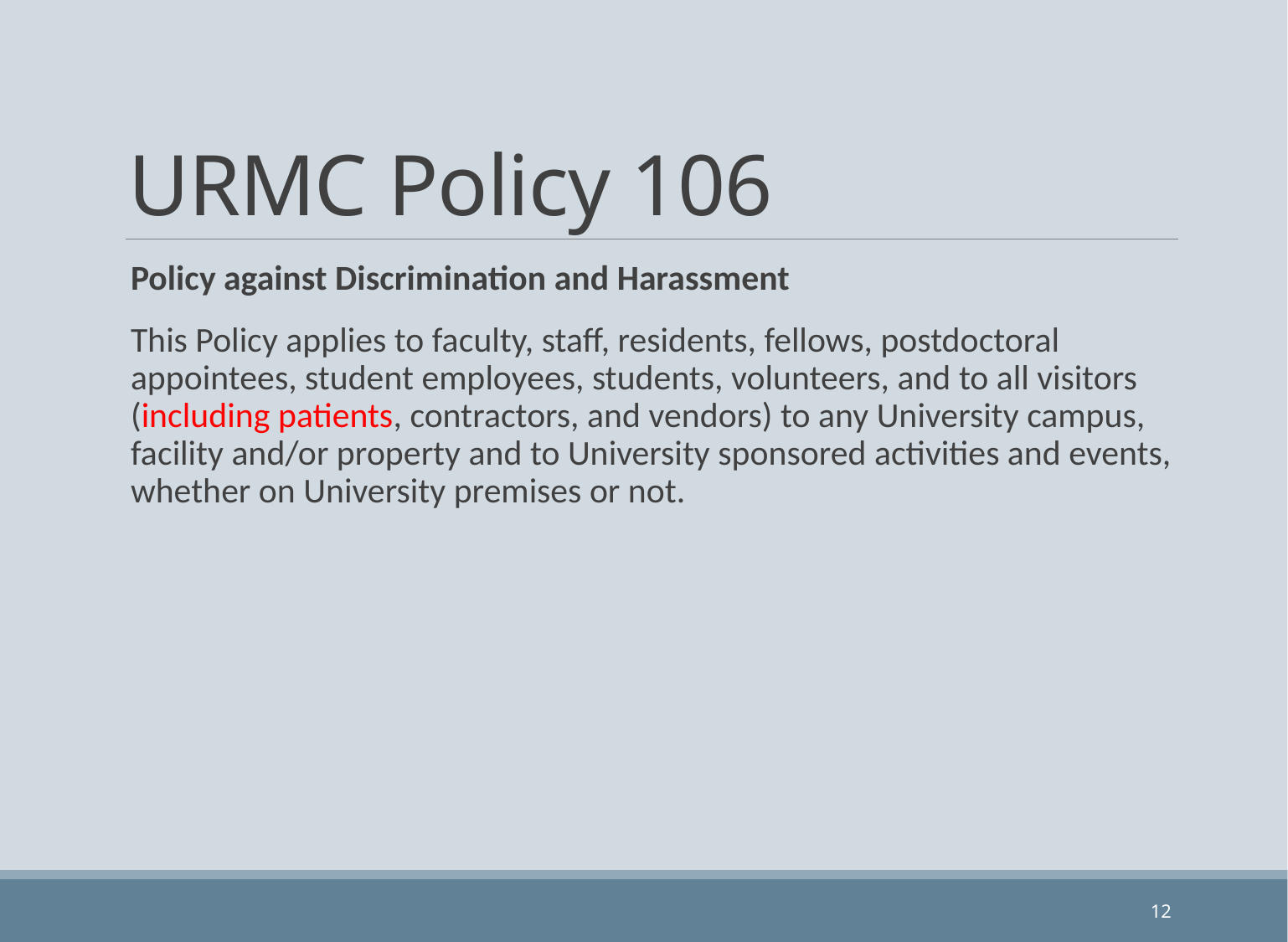

# URMC Policy 106
	Policy against Discrimination and Harassment
	This Policy applies to faculty, staff, residents, fellows, postdoctoral appointees, student employees, students, volunteers, and to all visitors (including patients, contractors, and vendors) to any University campus, facility and/or property and to University sponsored activities and events, whether on University premises or not.
12

## Slide 13
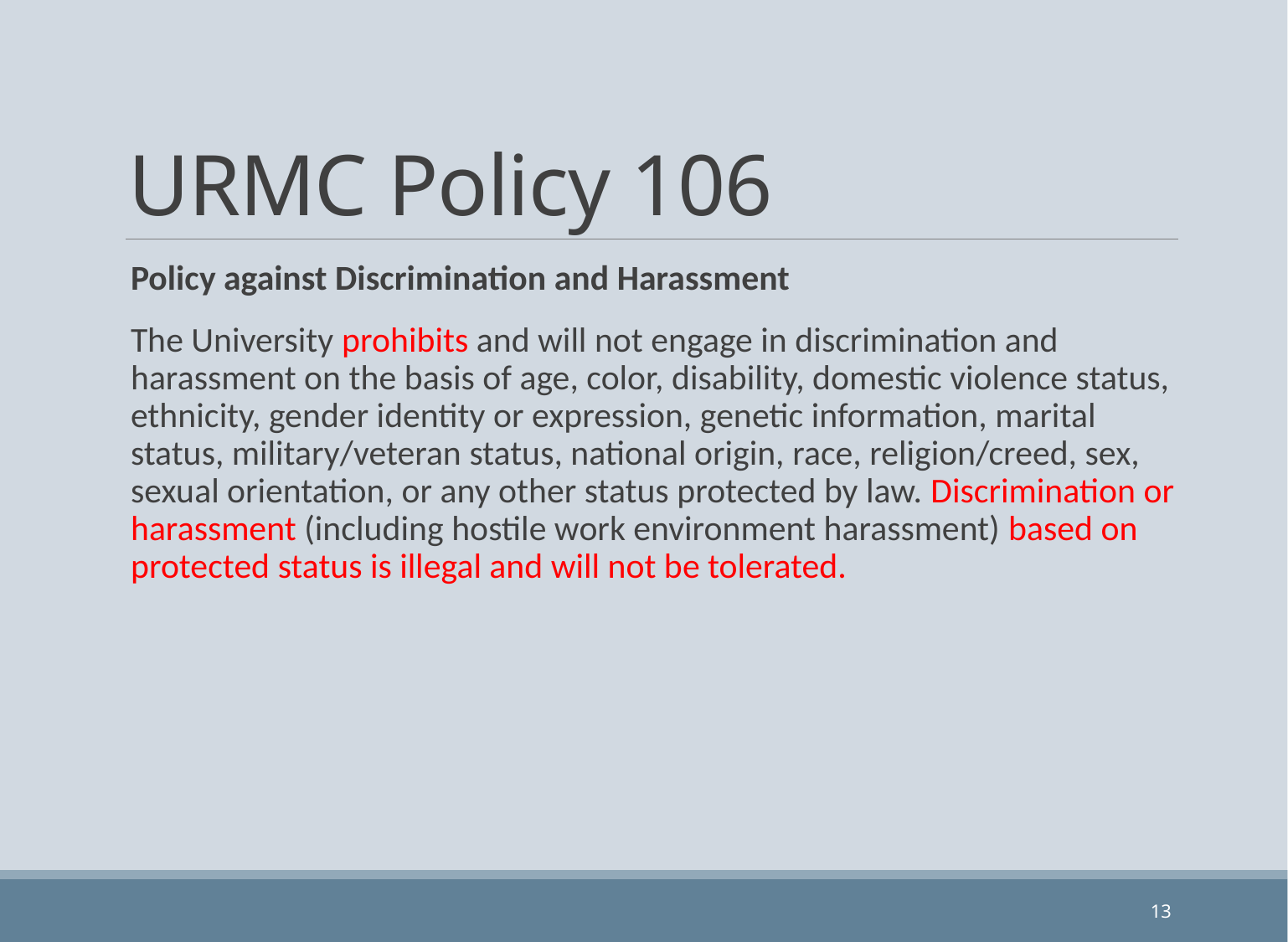

# URMC Policy 106
	Policy against Discrimination and Harassment
	The University prohibits and will not engage in discrimination and harassment on the basis of age, color, disability, domestic violence status, ethnicity, gender identity or expression, genetic information, marital status, military/veteran status, national origin, race, religion/creed, sex, sexual orientation, or any other status protected by law. Discrimination or harassment (including hostile work environment harassment) based on protected status is illegal and will not be tolerated.
13

## Slide 14
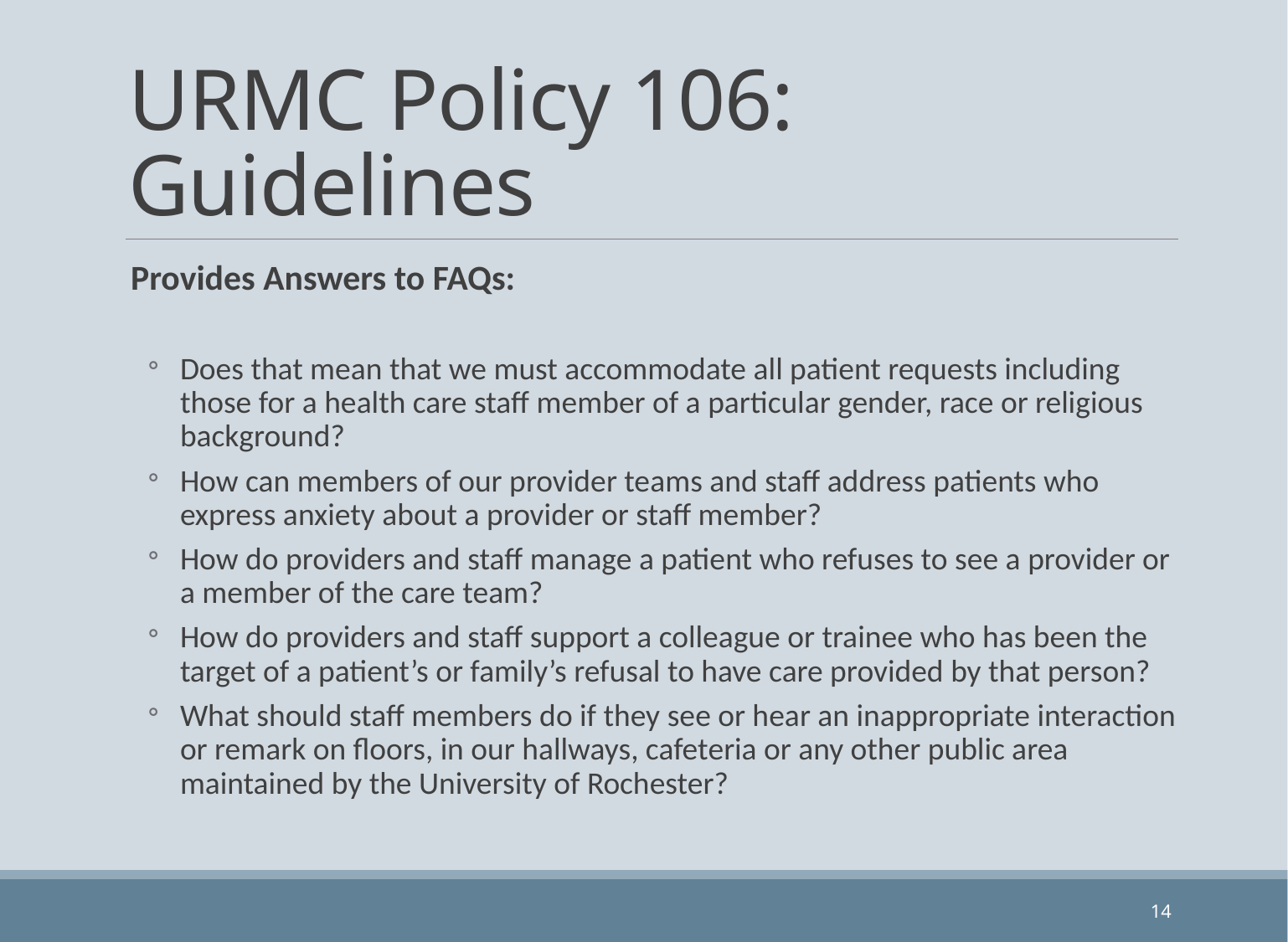

# URMC Policy 106: Guidelines
	Provides Answers to FAQs:
Does that mean that we must accommodate all patient requests including those for a health care staff member of a particular gender, race or religious background?
How can members of our provider teams and staff address patients who express anxiety about a provider or staff member?
How do providers and staff manage a patient who refuses to see a provider or a member of the care team?
How do providers and staff support a colleague or trainee who has been the target of a patient’s or family’s refusal to have care provided by that person?
What should staff members do if they see or hear an inappropriate interaction or remark on floors, in our hallways, cafeteria or any other public area maintained by the University of Rochester?
14

## Slide 15
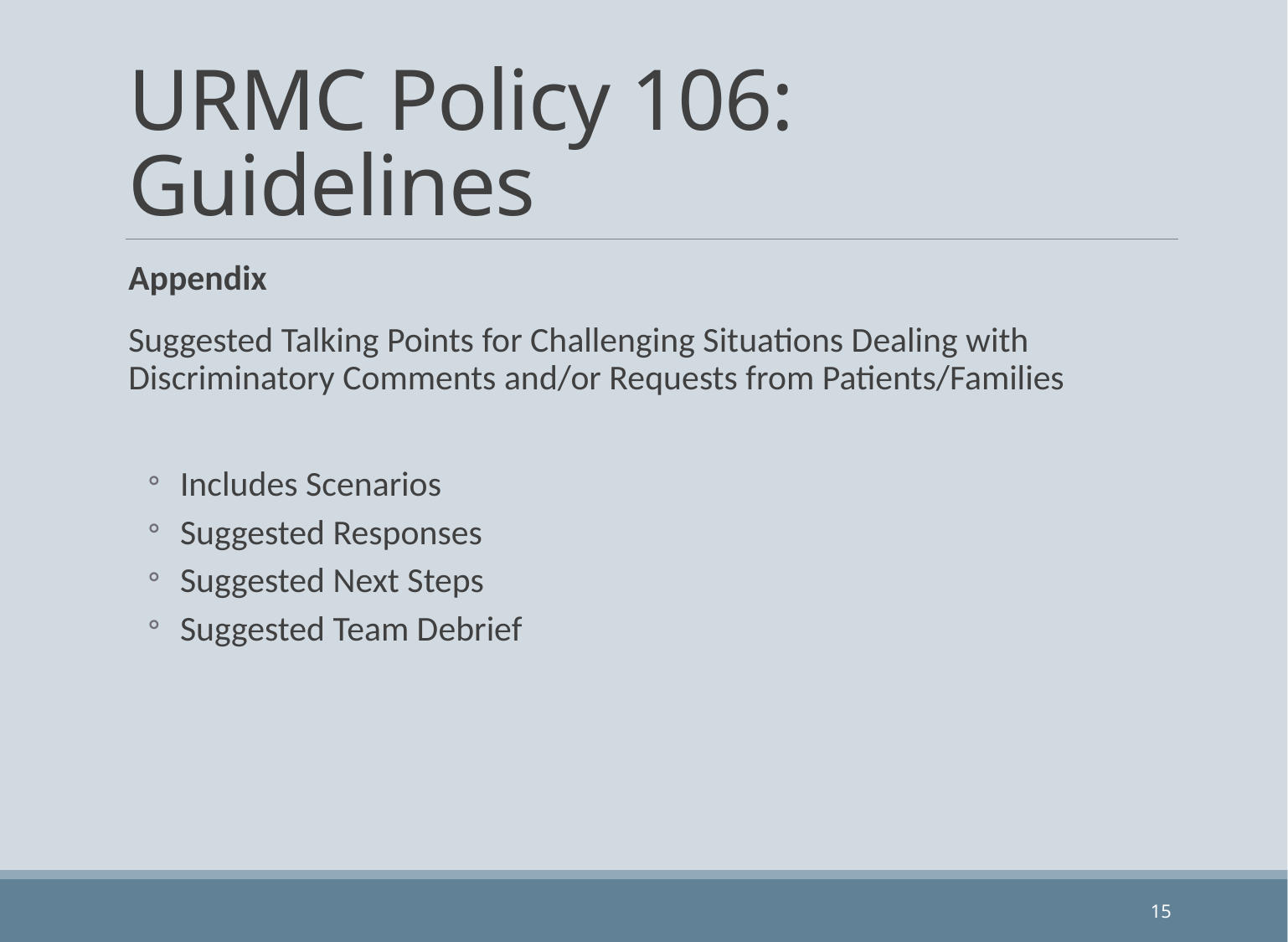

# URMC Policy 106: Guidelines
Appendix
Suggested Talking Points for Challenging Situations Dealing with Discriminatory Comments and/or Requests from Patients/Families
Includes Scenarios
Suggested Responses
Suggested Next Steps
Suggested Team Debrief
15

## Slide 16
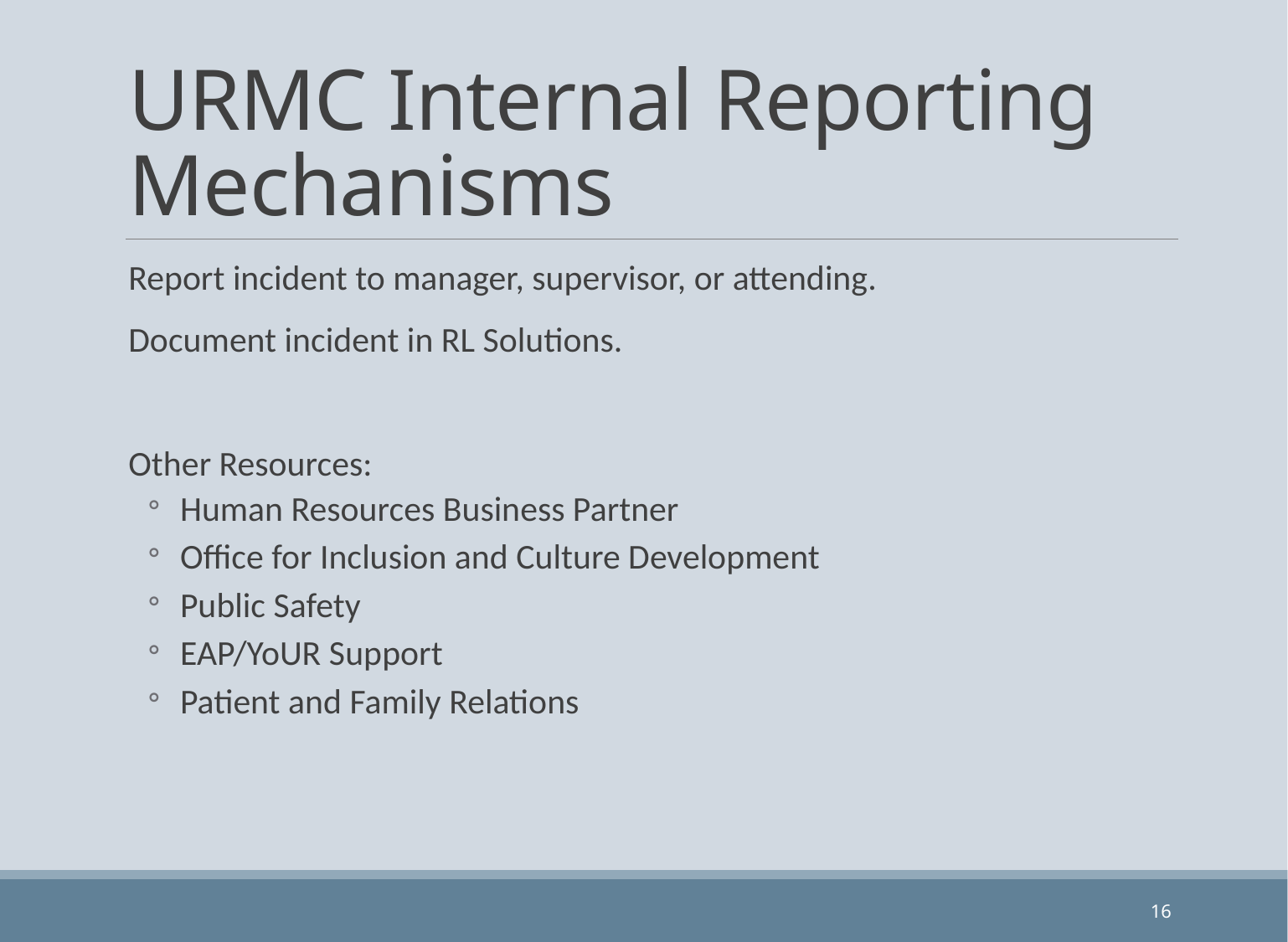

# URMC Internal Reporting Mechanisms
Report incident to manager, supervisor, or attending.
Document incident in RL Solutions.
Other Resources:
Human Resources Business Partner
Office for Inclusion and Culture Development
Public Safety
EAP/YoUR Support
Patient and Family Relations
16

## Slide 17
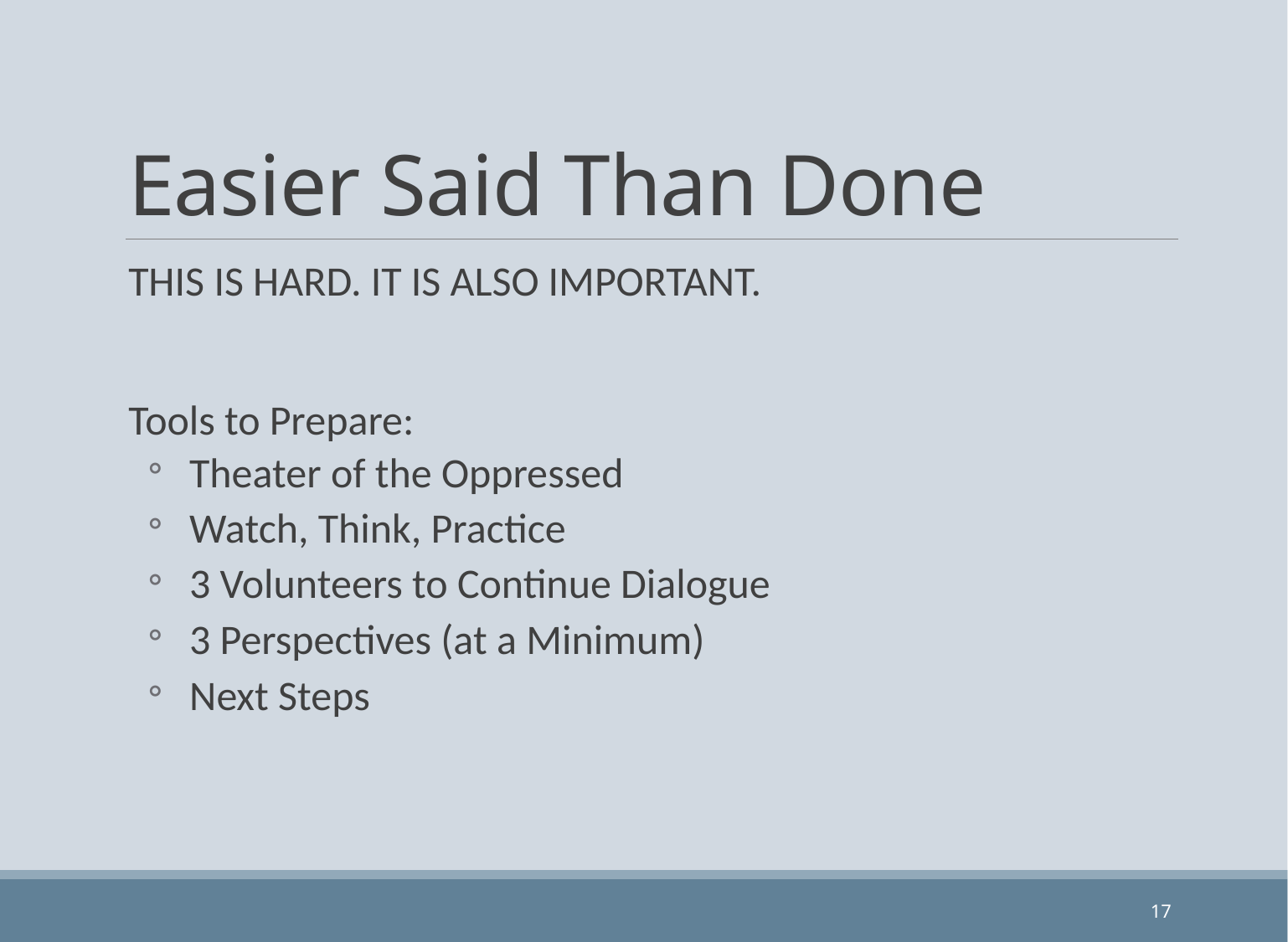

# Easier Said Than Done
THIS IS HARD. IT IS ALSO IMPORTANT.
Tools to Prepare:
 Theater of the Oppressed
 Watch, Think, Practice
 3 Volunteers to Continue Dialogue
 3 Perspectives (at a Minimum)
 Next Steps
17

## Slide 18
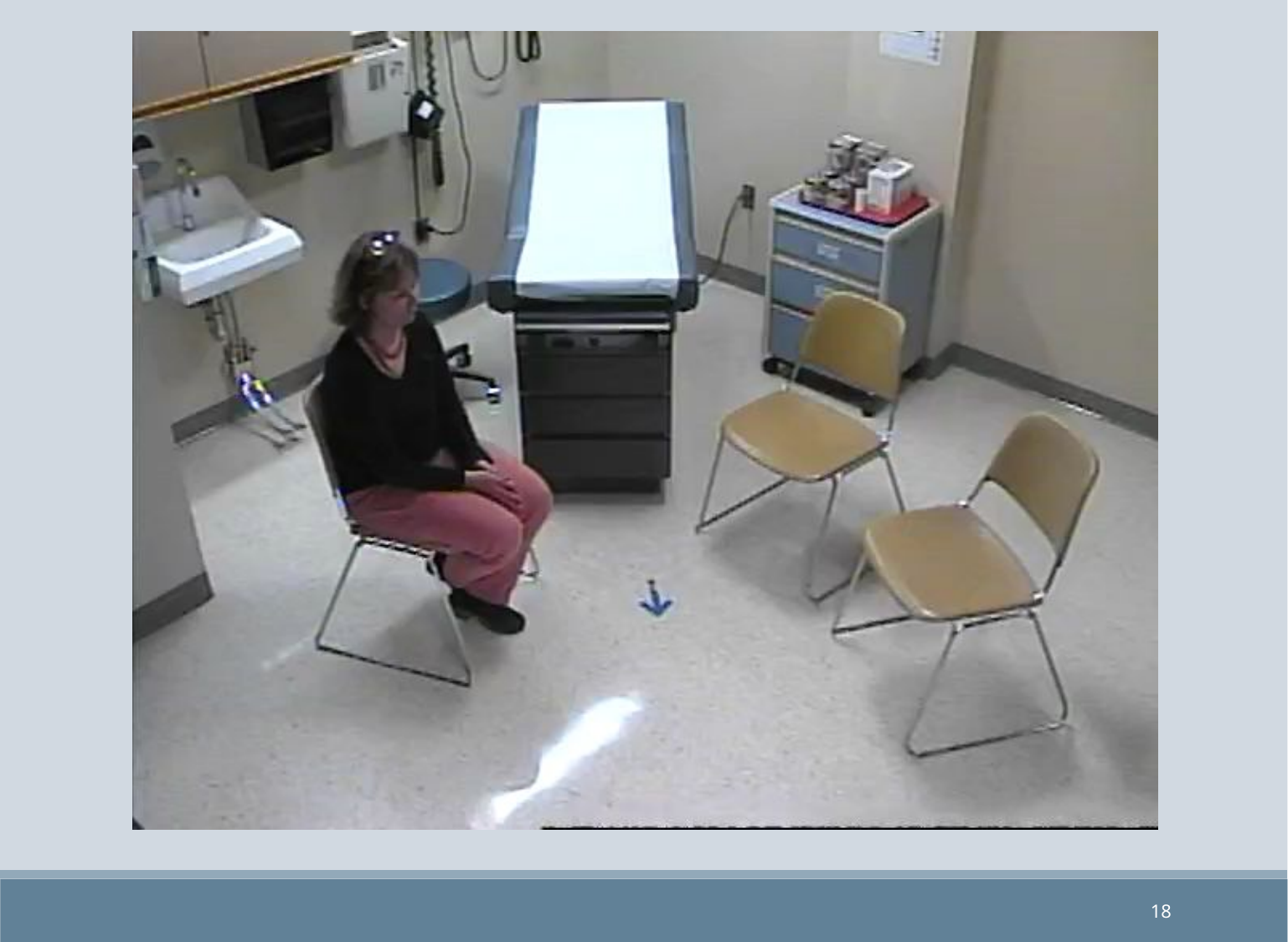

18

## Slide 19
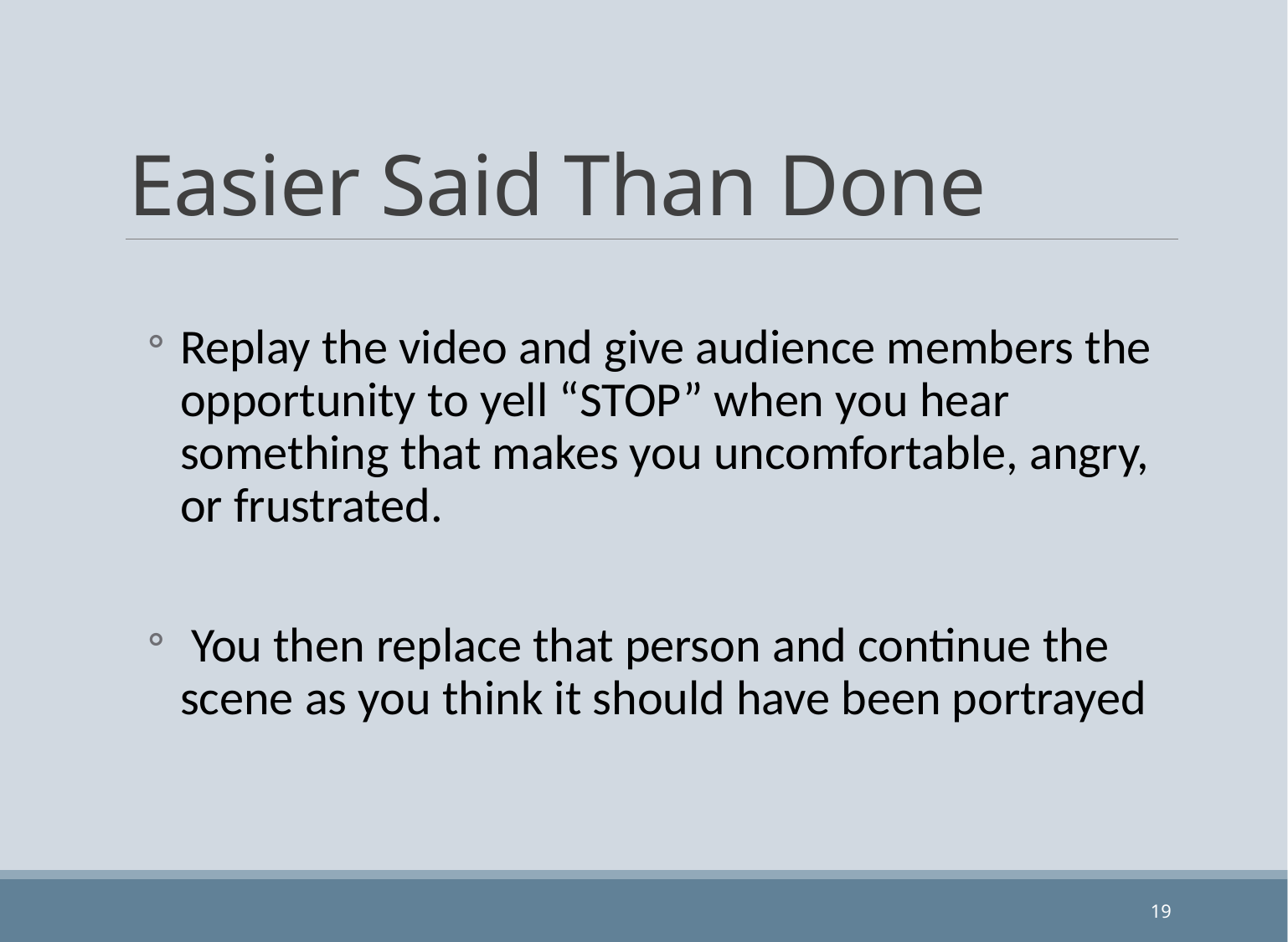

# Easier Said Than Done
Replay the video and give audience members the opportunity to yell “STOP” when you hear something that makes you uncomfortable, angry, or frustrated.
 You then replace that person and continue the scene as you think it should have been portrayed
19

## Slide 20
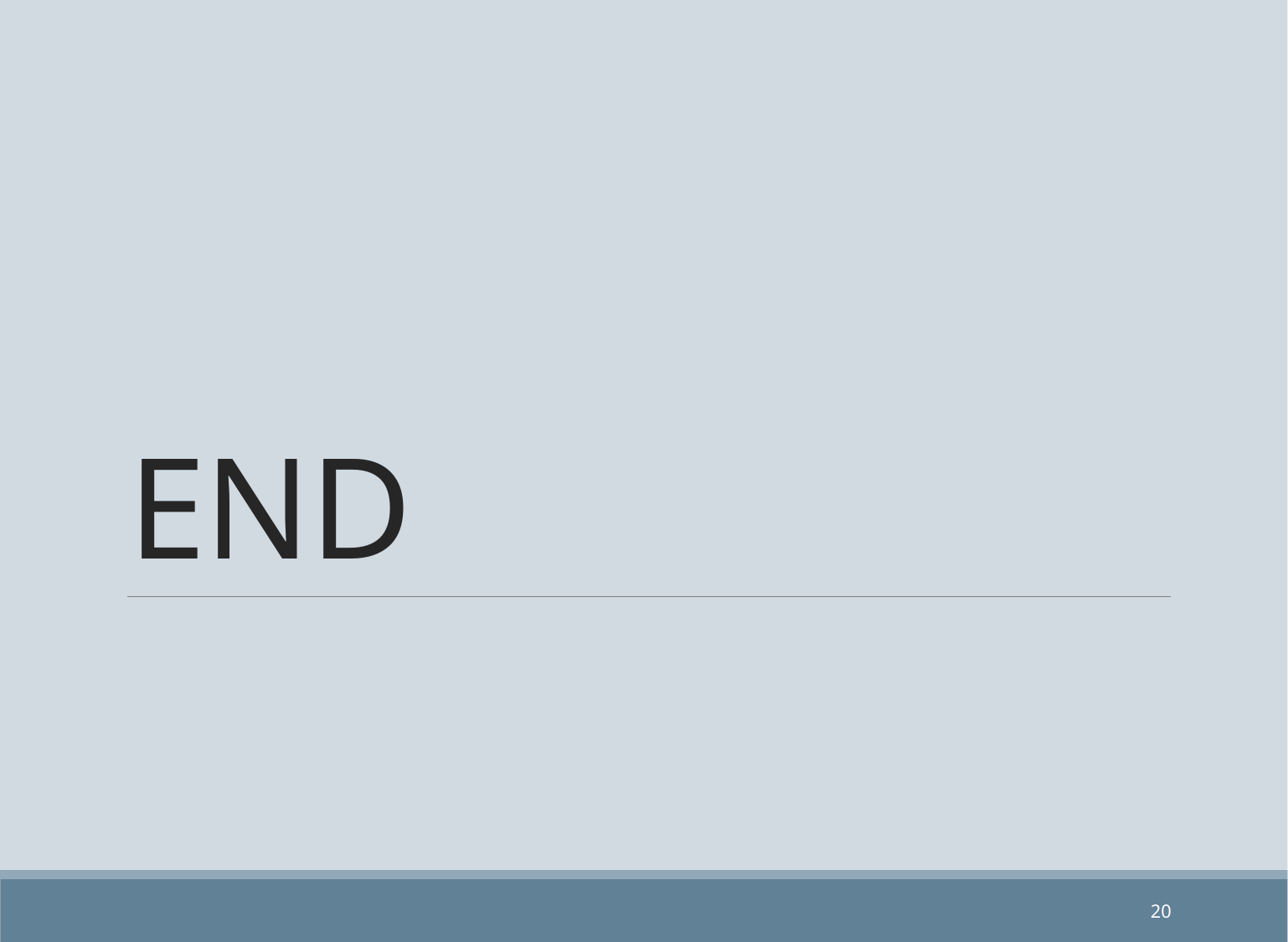

# END
20
