# Supplementary material for: Using Forum Theater as a Teaching Tool to Combat Patient Bias Directed Toward Health Care Professionals
Source: MedEdPORTAL. 2020 Nov 20;16:11022. doi: 10.15766/mep_2374-8265.11022 (PMC7678028; doi:10.15766/mep_2374-8265.11022)
Supplement: Supplementary file 1 — Presentation.pptxFacilitator Guide.docxPrefilmed Scenario.m4vEvaluation Form.docx [file mep_2374-8265.11022-s001.zip › B. Facilitator Guide.docx]

**Standard Facilitator Guide (90 Minute Session)**

*“Don’t touch me, you #@*#!”
Antidiscrimination Policies and Patient Biases: A Discussion*

| Activity | Time | Instructions |
| --- | --- | --- |
| Prepare the Room | In advance | (OPTION A) If the room has movable tables and chairs:   - Move the tables to the edges of the room to clear space. Place the appropriate number of chairs in a circle (one for each participant and for each facilitator) - Use the white board to write a welcome message   (OPTION B) If the room is an auditorium seating:   - Place three chairs in the front of the room - Use the white board to write a welcome message |
| Slide #1  Introductory Slide | 1 Minute | This is an introduction slide. Introduce the topic of bias and discrimination in healthcare, specifically the prevalence of bias and discrimination expressed by patients towards physicians. Begin to advocate for a cultural shift, towards protecting healthcare professionals from mistreatment, through the use of institutional policies and guidelines as well as the use of theater arts to provide a space to brainstorm and practice your ideas. |
| Slide #2  Disclosures and Agenda | 2 Minutes | This slide discloses any conflicts of interests and presents an agenda; some may prefer to separate this into two slides. This is the opportunity to begin to set the stage. We remind participants that we bring our own perspectives into the room; what is important to us is that we learn from each of these conversations. We encourage participants to critically evaluate the session. Be specific. Don’t just provide a high or low rating, tell us specifically why you chose a specific rating. Be critical and discerning. We use this information to improve our programming. |
| Slide #3  Learning Objectives | 5  Minutes | This slide describes the learning objectives for the session. Spend time going through each learning objective in order to set the scene for the remainder of the session. |
| Slide #4  Expectations for this Session | 10 Minutes | This slide describes the expectations for the session. Spend time going through each expectation in order to set the scene for the remainder of the session.   - Arrive at the beginning of the session and stay until the end. - Be responsible for your own well-being. Check your assumptions. - Notice and Suspend judgment, especially toward yourself. Focus on the process of observing and feeling rather than on assigning value. Step up and step back. - Confidentiality – What happens here stays here, What is shared here stays here, BUT … What’s learned here leaves here. - Challenge the idea not the person – “Let’s talk about that idea some more. I find that thinking to be flawed/problematic/harmful because” … Share your thinking and ideas not just judgment. - Meet people where they’re coming from – we’re all on a journey, in our learning process. Use “I” statements, and importantly, trust your body.   Ask the group if they would like to add any other expectations! |
| Slide #5  Personal Professional & Practice | 5 Minutes | The referenced article describes our approach to teaching ethics. We use the Personal, Professional, Practice framework. Aristotle’s General Moral Theory provides the foundation for this framework: "Aristotle asserts a *unity of virtue* thesis, which holds there are interdependencies between the possession of good judgment and the possession of moral virtues." Intellectual virtues are the "capacities or powers of understanding, judgment, and reasoning which enable the rational parts of the soul to attain truth (VI.2 1139bl 1-13)...." Moral virtues are the dispositions to act as reason dictates. The two are completely intertwined - how one feels in response to a situation will dictate how one sees the relevant features, which will influence how one acts. Today we are focusing on the professional and the practice, but knowledge of your own personal biases and values and the ability to consider the biases and values of others is foundational. As we discuss examples of explicit bias, please consider your own beliefs, values, and biases, and how those influence your decision-making process. |
| Slide #6  Case History of Dannie Abse | 10 Minutes | Those of you who provide care routinely face indirect insults, implicit bias, and the hateful, disrespectful patient. Providing care under those circumstances raises important challenges that we will not discuss today. If you want us to have dedicate time to that topic, we can schedule a different session. This session addresses explicit bias, and so our goal today is to provide the knowledge and the opportunity to practice the skills for your response to explicit bias and prejudice by patients (or family members) in the hospital. Describe the effects of implicit and explicit bias. Distinguish the role of the patient (personal) with the role of the provider (professional). Identify strategies for coping with explicit bias, including humor.  Here are two questions and answers from a NY Times interview with, Kumail Nanjiani, who write the screenplay “The Big Sick” with his now wife Emily Gordon. The movie was one of the big indie hits of 2017:  One of the things that was so refreshing about the movie is how you were one step ahead of the racist hecklers in the audience. How did you get to that point?  “Go back to ISIS,” that thing? I’d been heckled like that a few times in my stand-up, and at some point I realized, “This is happening enough that I can’t be rattled by it.” Here’s the problem. When someone’s racist to you, even though you know it’s their fault, it still hurts your feelings. It still flattens you. It still reduces you. You still feel weirdly bad about yourself. Onstage when you really need to be in control, when someone heckles you, it’s a big loss of control. So I had anticipated the lines based on what people had yelled at me before, and I’d written comeback lines.  What was your response again?  The response was, “That’s right, I am a terrorist. I just do stand-up comedy on the side to keep a low profile.” I had others. Before Osama [bin Laden] was caught they would yell, “Where is Osama?” And I would be, like, “I don’t know; he hasn’t texted me in forever.” |
| Slides #7-11  Personal Anecdotes and Federal Guidelines | 8 Minutes | These slides include personal anecdotes and specific occurrences of bias and discrimination targeting healthcare professionals. Use these slides to open up a discussion among participants who may have experienced or witnessed bias ad discrimination in your own institution. These slides also introduce the existence of federal guidelines for Title VII and historical legal cases, which together protect the rights of workers against bias and discrimination. |
| Slides #12-16  Institutional Policies, Guidelines, and Reporting | 10 Minutes | These slides discuss your institutional policies and guidelines related to anti-discrimination. Each individual institution will have their own related policies and guidelines, which should be added here. Each individual institution should also have unique reporting mechanisms for use by employees who have experienced bias and discrimination. It is important for the facilitators to familiarize themselves with their institution’s policies, guidelines, and reporting mechanisms. |
| Slide #17  Easier Said Than Done | 5 Minutes | This slide is an introduction to the idea of roleplay and Forum Theater. Emphasize again that this is a safe space but not a comfortable space.    **Things to Consider as the Facilitator**  Responding to and dealing with the moral distress of interactions with biased patients is overwhelming. Often, we are unprepared with a response in real time. Clinicians who are on the receiving end of biased patients are looking for and need the support of colleagues as allies in the moment or after the incident. Theatre of the Oppressed is a way for clinicians to respond in the moment and for their colleagues to step up as allies. It also ensures that individuals are knowledgeable of anti-discrimination and harassment policies so they can confidently address biased behavior from patients.  Theatre of the Oppressed (T.O.) is a form of popular community-based education that uses theater as a tool for social change. Originally developed out of Boal’s revolutionary work with peasant and worker populations in Latin America, it is now used all over the world for social and political activism, conflict resolution, community building, therapy, and government legislation. It is also practiced on a grassroots level by community organizers, activists, teachers, social workers, cultural animators, and more.  TO is a problem-solving technique in which an unresolved scene of oppression is presented. First, we want you to watch the video and notice all the moments that cause a gut reaction. We will then restart the video and give anyone in the audience a chance to yell STOP in the moment you feel frustrated or mad or angry and replace an actor you feel is being oppressed, struggling, or lacking power and improvise alternative solutions. This structure can be used to explore past and current situations, or as a “rehearsal for the future”.  We believe an integral part of this experience is to debrief with your colleague to see how she or he is doing after an incident of bias. It is important to see the event from his or her perspective and to get feedback from your colleague on if they felt supported and if there are things you can do in the future.  **Guidance for the Facilitator**  1) TO is dialogic and not didactic; it is promoted by asking questions rather than giving answers  2) TO requires the transformation of passive spectators into actors in order to initiate the changes  3) TO is moved from the individual to the general when participants perform in realistic scenarios  4) TO is about systems with widespread mass effects, and not about “good “people or “bad” people  5) TO is democratic such that the power is constantly being leveled horizontally by team members  - This is not a seminar  - This is not a standardized patient  - This is not just interactive theater  - This is learning and listening and discovering    Remember: “Being an oppressor or being oppressed is not a question of individual choices, nor is it a moral question. It is also not a question of essences: no one is oppressed or an oppressor in the essence of their nature. Rather, there are social groups that relate one to another. It’s a historical question. Boal states, “the question that ought to be asked about slavery is not whether the master is good or not, but rather, why does slavery exist?”    **Standards of Conduct for the Facilitator**  1) Facilitators must avoid all actions which could manipulate or influence the audience.  They must not draw conclusions which are not self-evident.  They must always open the possible conclusions to dialogue, stating them in an interrogative rather than an affirmative form, instead of being confronted with the facilitator’s own personal interpretation of events.  2) The facilitator must constantly be relaying doubts back to the audience so that it is they who make the decisions.  Does this particular solution work or not?  How has the oppression changed?  Is this realistic within your context? And this principle applies most of all in relation to the spect-actors’ interventions.  Often a spect-actor will say ‘Stop!’ before the preceding spect-actor has finished his/her own intervention.  The facilitator must then tactfully persuade the newly intervening spect-actor to exercise patience, while also trying to sense what the audience wants; they may well have already understood the intervention and want to move on.  Once again, the decision rests with the audience.  3) Facilitators must watch out for all ‘magic’ solutions.  They can interrupt the spect-actor/protagonist’s action if they consider this action to be magic, not ruling that it is magic, but rather asking the audience to decide.  *We should take note of the fact that when the audience shouts that such-and-such a solution is not magic, that the solution is possible, that shout is the beginning of a process of self-motivation on the spect-actor’s part, it is the stimulus for real action.  Sometimes the solutions proposed are at the opposite end of the spectrum to ‘magic’, they are inadequate.  In these cases, the facilitator must try to push the spect-actors into finding more active solutions.  The magic solution is cheating, but the inadequate solution is demobilizing. 4) The physical stance of the facilitator is extremely important.  Some facilitators are tempted to mix with the audience, to sit with other spect-actors; this can be completely demobilizing.  Others, by their demeanor, allow their own doubts, their own indecision or timidity, to show through.  Now everything that happens on stage, by which I mean all the images produced by the body or by objects, is significant.  If the facilitator on stage is tired or confused, he or she will transmit a tired and disoriented image to the audience. But beware – being dynamic does not mean seeking to influence the outcome! 5) Finally, the facilitator must be Socratic – dialectically, and, by means of questions, by means of doubts, she or he must help the spectators to gather their thoughts, to prepare their actions.  The facilitator is a midwife.  The facilitator must assist the birth of all ideas, of all actions.  Going further than Socrates, who framed questions that expected answers, and, in so doing, limited the field of questions: who do you want to talk about?  We try to avoid any form of manipulation of the participants.  **Helpful Hints for the Facilitator**  1) Be informed about the Issue!  Develop 8-10 questions before the performance that explain or address the issue. 2) Place yourself physically between the audience and actors (the facilitator is the literal and metaphysical “bridge”). 3) Always be engaged. Always be active. Don’t allow the energy to drop between the audience and the actors. 4) Use questions to stimulate discussion (Is this realistic? Does this happen in your community? Who is the oppressor? Who is the oppressed? How has the oppression changed after this spect-actor’s intervention?) 5) Thank the spect-actors for their ideas and interventions.  They are taking a risk to be on stage and it is the facilitator’s responsibility to thank each spect-actor. 6) Enter each interactive theatre experience with few or no expectations to the audience does not feel pressured by the facilitator’s anxieties, expectations, or nervousness. |
| Slide #18  Pre Filmed Video | 2 Minutes | Play the video for the group, and explain that three volunteers will be asked to reenact the scene. Ask for three volunteers and assign each a role – the protagonist (Dr. Khan), bystander (Dr. Brown), or the antagonist (patient). Reassure the volunteers they do not need to reenact the scene verbatim but capture the essence of the antagonist, protagonist, and bystander. The antagonist never changes their stance and cannot be swapped out during all interactions of the scene. Only the protagonist a bystander can be substituted by audience members to change the outcome of the scene and end the oppression experienced by the protagonist. It is important to remember that the goal is to have the protagonist and bystander change the outcome in a way that is *authentic* to them. It will be different for each individual.  *Part 1*  Introduce the scene and the backstory of the scene. Tell the audience to watch and notice parts of the interactions that make them feel uncomfortable. Start the scene with the audience participating in ‘1,2,3 ACTION!’. Completely run through the scene from start to finish without any interruptions. At end acknowledge actors with applause. |
| Slide #19  Video Scenario | 20 Minutes | *Part 2*  Audience interaction – ask them what they thought of the scene. Were there any scenes that they thought were difficult interactions? Thank the audience and tell them we are going to have the volunteer actors reenact the scene, but this time you want them to watch carefully and if at any point they see something that makes them feel uncomfortable and could change the way the protagonist (use character name) is acting, you want them to yell ‘STOP!’  **Practice with the audience yelling ‘STOP!’**  Restart the scene with the audience participating in ‘1,2,3 ACTION!’ When someone yells ‘STOP!’ – stop the performance. ***Note*** *– if the scene is continuing without anyone yelling stop, you STOP the scene and ask the audience were there no moments that made them feel uncomfortable*  Acknowledge the person who yelled stop and use the following dialogue:  What is your name? Why did you stop the performance? Discuss.  Can you tell us what you would do differently as the protagonist (use character’s name)?  Why don’t you come up and show us that? (Don’t ask if they feel comfortable coming up or if they would like to come up). Applause. When they come up to the stage, *they* *assume the character of the protagonist by name and also hand-off an item from the actor to spect-actor.* Ask the spect-actor at which part of the scene they would like to resume the performance.  Restart the play with the audience participating in ‘1,2,3 ACTION!’  Allow the scene to play out – stop if it is going nowhere and the spect-actor is not changing the dynamics. Applause.  Approach spect-actor and use the following dialogue:  ‘Do you think you accomplished what you set out to do?’ Discuss.  It is important to keep affirming the spect-actor in the debrief. Thank them for participating and ask them to return to their seat BEFORE engaging audience in discussion. Ask the audience what they think the spect-actor accomplished and try not to open the door to any criticism of the spect-actor and their performance. If someone does criticize – ask them to explain what they would do. After they explain say “Why don’t you come up and show us that?” |
| Slide #20  End of Session | 12 Minutes | Ending the Session  After a number of iterations and when the facilitator feels all possibilities are played out (around 5-6 times) ask the audience to reflect on the scenes. Were there scenes that were more effective than other? Which scenario did they think they could realistically do during a patient interaction? Ask if this was helpful? If so, why? If not, why not?  Thank them for their bravery and courage. End the session with an appreciation circle (Have people stand in a circle). Ask people to give one line or word about what they learned and will take away from the session.  End Session |
