# Supplementary material for: Using Forum Theater as a Teaching Tool to Combat Patient Bias Directed Toward Health Care Professionals
Source: MedEdPORTAL. 2020 Nov 20;16:11022. doi: 10.15766/mep_2374-8265.11022 (PMC7678028; doi:10.15766/mep_2374-8265.11022)
Supplement: Supplementary file 1 — Presentation.pptxFacilitator Guide.docxPrefilmed Scenario.m4vEvaluation Form.docx [file mep_2374-8265.11022-s001.zip › D. Evaluation Form.docx]

**Antidiscrimination Policies and Patient Biases: A Discussion**

Please indicate your professional category:

__Physician __Registered Nurse __Physician Assistant __Staff
__Resident/Fellow __Nurse Practitioner __Psychologist __PhD/Researcher
__Medical Student __Nursing Student __Social Worker __Other

Please indicate your opinion of these objectives:

As a result of attending this educational activity, I believe that I am able to …

1. Examine their own beliefs and values related to bias and discrimination that is directed at healthcare professionals. __Yes __No

2. Describe the impact of racial bias and racial discrimination on healthcare professionals. __Yes __No

3. Appraise the relationships between law, policy, and institutional culture around diversity and inclusion. __Yes __No

4. Apply knowledge of law and policy, as well as communication skills practiced, in the adaptation of Forum Theater to promote a culture of diversity and inclusion. __Yes __No


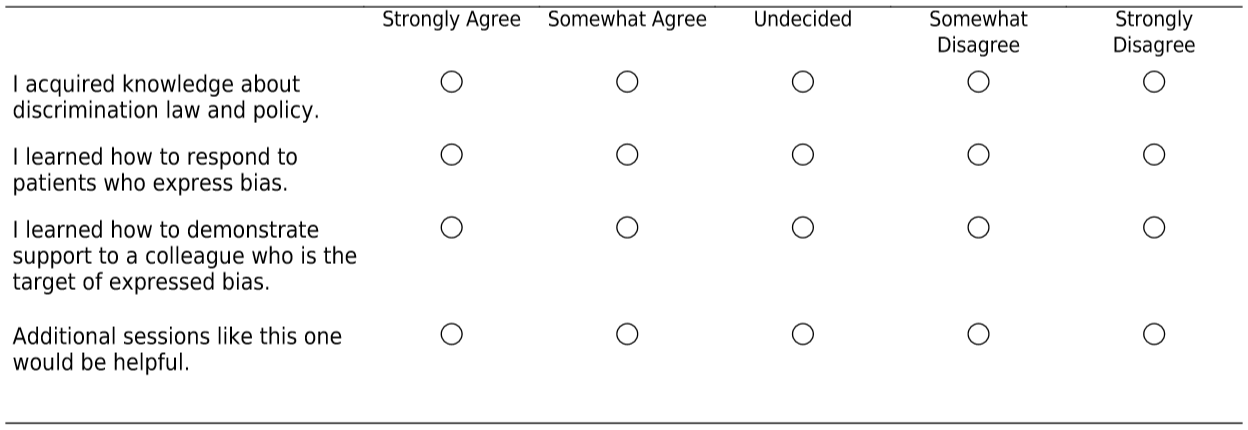

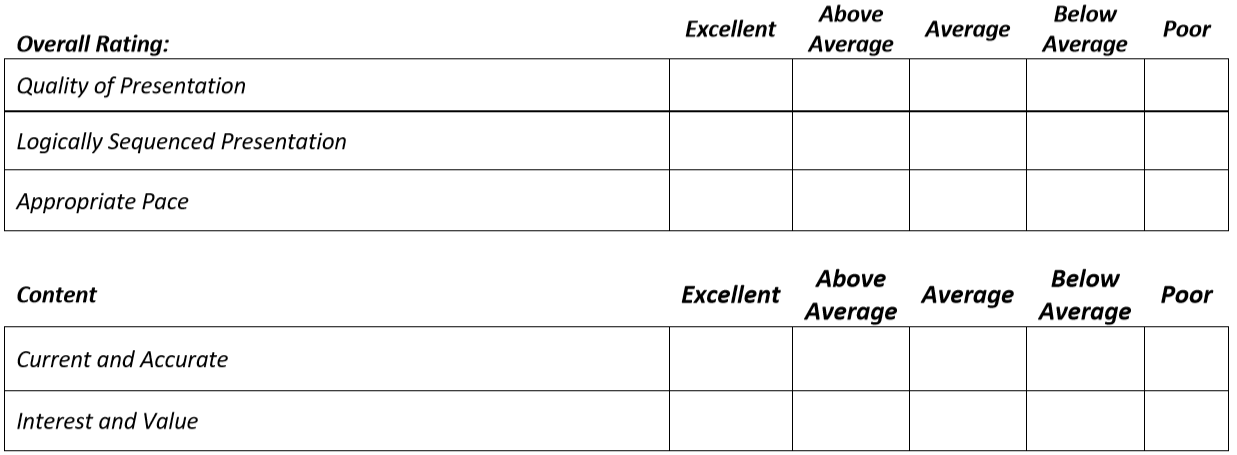
Please indicate your impression of this session:

1. How can we improve the educational content of this session?

2. How will you incorporate the information learned at this session into your teaching, research, or practice?

3. General Comments:
