# Supplementary material for: Using Forum Theater as a Teaching Tool to Combat Patient Bias Directed Toward Health Care Professionals
Source: MedEdPORTAL. 2020 Nov 20;16:11022. doi: 10.15766/mep_2374-8265.11022 (PMC7678028; doi:10.15766/mep_2374-8265.11022)
Supplement: Supplementary file 1 — Presentation.pptxFacilitator Guide.docxPrefilmed Scenario.m4vEvaluation Form.docx [file mep_2374-8265.11022-s001.zip › _Educational_Summary_Report_11022.pdf]

MedEdPORTAL® The AAMC Journal of Teaching and Learning Resources

Original Publication Open Access

Using Forum Theater as a Teaching Tool to Combat Patient Bias Directed Toward Health Care Professionals

Nina Rizk, MPH, MS*, Shaunpaul Jones, Margie Hodges Shaw, JD, PhD, HEC-C, Adrienne Morgan, PhD

*Corresponding Author: Nina_Rizk@urmc.rochester.edu

Abstract

Introduction: Health care professionals who identify as members of underrepresented and racial minority groups may experience bias from patients and patient families. These occurrences disrupt the educational and therapeutic environments, distress the targeted individuals and allies, and create potential legal liability. Yet there are few educational opportunities for individuals to brainstorm and implement strategies for responding professionally during such instances. **Methods:** Presented first as a grand rounds, then an invited workshop, and finally an invited series, this educational activity was developed in a stepwise manner over the course of a year. Each format was sequentially modified based on feedback from participants—more than 200 physicians and other health care professionals—using evaluation forms that were voluntary and anonymous. The educational activity used an adaptation of forum theater, in which participants role-played an instance of oppression with a goal of altering the ultimate outcome. This approach provided participants with the opportunity to develop and rehearse responses to workplace bias in a way that preserved the provider-patient relationship. **Results:** Feedback for these educational sessions was overwhelmingly positive. Participants noted the importance of acknowledging and addressing bias in the workplace and encouraged facilitators to expand the sessions in length, frequency, and scope. **Discussion:** Forum theater is a methodology that can be employed in health care to teach appropriate and authentic responses to expressed patient bias while maintaining the therapeutic relationship. The positive reception from participants in our preliminary sessions established a strong foundation for future improvements to this work.

Keywords:
Faculty Development, Theater, Bioethics, Bioethical Issues, Institutional Culture, Humanities (Art, Literature, Music), Diversity, Inclusion, Health Equity, Anti-racism, Editor's Choice

Educational Objectives

By the end of this activity, learners will be able to:

1. Examine their own beliefs and values related to bias and discrimination directed at health care professionals.
2. Describe the impact of racial bias and racial discrimination on health care professionals.
3. Appraise the relationships between law, policy, and institutional culture around diversity and inclusion.
4. Apply knowledge of law, policy, and institutional culture, as well as practiced communication skills, in the adaptation of forum theater to promote a culture of diversity and inclusion in the workplace.

Citation:
Rizk N, Jones S, Shaw MH, Morgan A. Using forum theater as a teaching tool to combat patient bias directed toward health care professionals. *MedEdPORTAL*. 2020;16:11022.
https://doi.org/10.15766/mep_2374-8265.11022

Introduction

The physician workforce is not immune to the epidemic of bias and discrimination that runs rampant in the United States and throughout the world. A national study conducted in the United States showed that 59% of physicians surveyed had experienced bias from patients.[1] A longitudinal study conducted in Canada found that 45% of family medicine residents reported intimidation, harassment, and/or discrimination from patients while in the workplace.[2] March and colleagues surveyed pediatric residents about the prevalence of discriminatory comments in the workplace as part of a curricular activity to teach communication skills, and 56% of the residents reported experiencing discrimination from patient families.[3] Hu and colleagues completed a national cross-sectional survey of general surgery residents linking high rates of discrimination and abuse to burnout and suicidal thoughts.[4] These encounters primarily centered on visibly apparent characteristics related to gender, ethnicity, national origin, race, and religion.[1-4] These

Copyright © 2020 Rizk et al. This is an open-access publication distributed under the terms of the Creative Commons Attribution license.

studies lend evidence to the claim that physicians frequently experience bias from patients and patient families.

The most compelling evidence of workplace discrimination arises from personal anecdotes published by health care professionals who have experienced this mistreatment firsthand.[5,6] Unfortunately, despite this evidence that workplace bias and discrimination exist, there is limited information that individuals (targets) and their colleagues (bystanders) can use to develop and practice strategies for how to respond to patients and patient families who exhibit such behaviors. Current literature describing strategies for responding to these patients focuses on two types of potential solutions. The first solution is systemic, with recommendations for medical institutions to compose and implement specific policies and guidelines condemning bias and discrimination against health care professionals on the basis of race and other factors.[7,8] The second solution is individual, with descriptions of how a provider might respond to a patient who exhibits biased or discriminatory behavior while maintaining the provider-patient relationship.[7,8]

While systemic policies are necessary and individual guidelines are important for combating workplace bias, the ability to appropriately respond to such a patient also requires a meaningful understanding of one's own values and beliefs. Aristotle's general moral theory supports this ethical education; Aristotle asserts the unity of virtue. Intellectual virtues, or the ability to reason, and moral virtues, or the disposition to act as reason dictates, are inextricably intertwined.[9] Developing the reasoning skills and good judgment necessary for right action requires practice, and culture change requires group action. Effectively confronting patient bias in the workplace mandates this critical component: a formal training for health care professionals involving active practice and collaborative action so as to accurately identify and constructively respond to patients expressing bias within the workplace. While this literature is beginning to develop, it remains insufficient.[10,11]

Theater is a uniquely effective conduit by which medical education and medical training can be delivered and shows great potential in promoting cultural change.[12-19] This is particularly due to the ability of theater to increase engagement, interactivity, and comfort, while also allowing for the discussion of sensitive topics.[20] Forum theater (FT)—based on Paulo Freire's Pedagogy of the Oppressed[21] and Augusto Boal's Theater of the Oppressed[22]—is a method that has shown much promise in stimulating deliberation on issues surrounding bias expressed by patients and family members. FT describes a method of acting in which participants portray different roles in order to confront their experiences with oppression through the use of creative expression and team collaboration. FT was initially intended to be conducted exclusively by the oppressed, for the oppressed, such that the individuals who experienced oppression were the individuals generating the solutions for confronting the oppression. However, the members of health care teams—including students, residents, physicians, advanced practice providers, nurses, social workers, administrators, and so on—vary in their relationships with oppression, with some members experiencing oppression and others perpetuating it, either directly through words and actions or indirectly through passive silence. FT in the health care setting is therefore intended both to provide oppressed individuals with strategies for responding to patients who express bias and to provide their colleagues with strategies for supporting the oppressed individuals.

There are typically three primary actors in FT: (1) a protagonist who is experiencing oppression, (2) an antagonist who is perpetuating oppression, and (3) bystanders who are witnessing oppression.[22] The protagonist can represent any health care professional who is experiencing oppression in the form of explicit racial bias and/or discriminatory comments. The goal of the actor portraying the protagonist is to alter the ending of the scenario and break the cycle of oppression. The antagonist can represent any patient or patient family member who is perpetuating oppression in the form of explicit racial bias and/or discriminatory comments. The goal of the actor portraying the antagonist is to maintain the ending of the scenario and continue the cycle of oppression. Finally, the bystanders can represent any others present who are failing to respond appropriately to the oppression being witnessed. The goal of the actors portraying the bystanders is to alter the ending of the scenario and break the cycle of oppression, doing so by working as an active and supportive ally for the protagonist.

FT is further characterized by audience participation in the scenario as audience members transition from their roles as spectators to their roles as *spect-actors.* These spect-actors first observe and then participate in the scene, inserting themselves into the role of either the protagonist or the bystander in an effort to change the dynamic with the antagonist and alter the ultimate outcome of the scene to break the cycle of oppression. The antagonist never changes course; the power of this approach is that the protagonist and the bystander are responsible for changing the encounter, and potentially the group relationships, by altering their responses to the expressed bias.

Most FT participants find talking about bias and discrimination uncomfortable. However, providing health care professionals

Copyright © 2020 Rizk et al. This is an open-access publication distributed under the terms of the Creative Commons Attribution license.

with opportunities to practice these difficult conversations in a simulated environment allows for experimentation with a wide array of approaches. Engaging in this work with colleagues allows for the consideration of the various perspectives and experiences of all team members and reinforces cultural expectations around inclusion and diversity. The ultimate goals of these sessions are for participants to leave with a greater appreciation of the impact of bias and discrimination on individuals and culture, strategies for addressing patient expressions of bias and related actions, and motivation to advocate for culture change within their respective workplaces.

This approach to professional education—the use of playacting to reenact encounters with oppression in the workplace—is itself not a novel idea. However, its application in the health care setting in particular is truly one of a kind. Using this modified version of FT, health care professionals are able to work with one another to simulate an encounter with a patient who expresses bias and to brainstorm feasible solutions for how to respond to the expression of words and actions based in bias and discrimination. The publication of this innovative method of education is intended to add to the limited literature currently available to health care professionals who witness or experience expressed racial bias. It does so using a unique and multipronged approach in which facilitators go beyond the mere provision of information about institutional policies and guidelines, by framing the strategy within an ethical framework and forming a collaborative space in which participants attempt to cite and enforce these institutional policies and guidelines in a role-play scenario based in reality.

Methods

This intervention began as an educational activity and not as a research project, and so, each initial session was followed by the dispersal of the standard evaluation form required for all continuing education credits at the institution. We initially reviewed the evaluation forms in order to improve subsequent sessions, and with the feedback provided by participants, the sessions began to develop so as to better fit the target audience and stated goals. This section describes the iterative process by which the educational activities evolved, and identifies key themes useful for other institutions in their implementation of FT.

The University of Rochester School of Medicine and Dentistry (URSMD) Offices for Medical Education and Division of Medical Humanities and Bioethics collaborated with the University of Rochester Medical Center (URMC) Office for Inclusion and Culture to adapt FT for use in the health care setting. Over the course of a year, the educational sessions took on a variety of forms, which were implemented in a stepwise manner and sequentially modified based on feedback from participants and the unique needs of each participant group. Following the initial introduction of FT in the format of a grand rounds advertised to members of the URMC and URSMD community, the URMC residency program directors for the internal medicine (IM) and emergency medicine (EM) departments requested sessions for residents in their respective programs, and the Department of Psychiatry requested sessions for the department's Summer Education Series open to all community members at URMC (Table 1).

Following the initial grand rounds and in response to requests for additional educational activities using FT, the facilitators realized that they would benefit from expert training and therefore invited Katherine Burke, MFA, project coordinator, The Art and Practice of Medicine, Lerner College of Medicine, to Rochester to provide a 1-day training about Theater of the Oppressed and FT. They also attended an additional 3-day educational workshop to further develop their knowledge and skills related to FT. This workshop was offered by Carli Gaughf, MA, of the Applied Theater Center in Greenville, South Carolina, and focused on FT for marginalized communities. Finding this educational experience invaluable and the number of requests for training from the community increasing, the facilitators invited Ms. Gaughf to Rochester as an artist-in-residence. During her stay, she trained a troupe of group facilitators, ensuring the sustainability of this intervention and meeting the growing demand for this educational activity.

Based on lessons from these concentrated experiences and specific training in FT, the facilitators developed a collection of educational materials, including a set of presentation slides (Appendix A) and a corresponding facilitator guide (Appendix B). These resources were intended to serve as standard templates and have been adapted for each iteration of this educational intervention as appropriate, based on the length of the session, the target audience, and the stated goals. Each session was designed such that participants were not expected to have any awareness or understanding of this novel educational model prior to arriving at the sessions. In addition to the training in FT, the facilitators also had expertise in law, bioethics, education, diversity and inclusion, and specific institutional policies and guidelines.

Each session began with an introduction to the work of the Division of Medical Humanities and Bioethics and the framework supporting the teaching.[9,23] This involved a digital presentation for both the grand rounds and the invited workshops and an analogous verbal presentation for the invited series. This

Copyright © 2020 Rizk et al. This is an open-access publication distributed under the terms of the Creative Commons Attribution license.

Table 1. Stepwise Incorporation of Forum Theater at University of Rochester School of Medicine and Dentistry and University of Rochester Medical Center

Type	Department	Audience	Purpose	Duration
Grand rounds	University of Rochester Medical Center	Open to all University of Rochester Medical Center: general audience	Outline the institutional policies and guidelines.<br>Introduce the pedagogy and format of forum theater.	50-minute session, offered twice
Invited workshops	Internal Medicine	Resident physicians and physician leadership in resident education	Outline the institutional policies and guidelines.<br>Introduce the pedagogy and format of forum theater.<br>Encourage culture-specific playacting of encounters with patients who exhibit racial bias.<br>Brainstorm and practice realistic and achievable strategies for how to appropriately respond to patients who exhibit racial bias.	50-minute session, offered twice to accommodate all residents
Invited workshops	Emergency Medicine	Resident physicians and physician leadership in resident education	Outline the institutional policies and guidelines.<br>Introduce the pedagogy and format of forum theater.<br>Encourage culture-specific playacting of encounters with patients who exhibit racial bias.<br>Brainstorm and practice realistic and achievable strategies for how to appropriately respond to patients who exhibit racial bias.	50-minute session, offered twice to accommodate all residents
Invited series	Psychiatry	Interdisciplinary health team	Outline the institutional policies and guidelines.<br>Introduce the pedagogy and format of forum theater.<br>Encourage culture-specific playacting of encounters with patients who exhibit racial bias.<br>Brainstorm and practice realistic and achievable strategies for how to appropriately respond to patients who exhibit racial bias.	Seven 50-minute sessions

introduction prepared participants to expect a different kind of conversation, one that encouraged voices with differing perspectives and experiences.[23] Next, the facilitators briefly reviewed the literature on expressed bias in health care, federal and state law, and university policy and guidelines. The URMC policy against discrimination and harassment applied to all "faculty, staff, residents, fellows, postdoctoral appointees, student employees, contractors, students, volunteers, and visitors (including patients and their family members…)."[24] In compliance with federal law, this policy explicitly prohibits discrimination and harassment on the basis of a number of identifying factors, including race.

Simultaneously with the introduction of these educational sessions, the vice president of diversity and inclusion for URMC also oversaw the creation of institutional guidelines to support the application of existing policy within the clinical setting. These guidelines state that it is not the practice of URMC to "change providers or assigned staff when the request or statement is related solely to the provider's personal identity such as… race" in nonemergent situations.[25] Although the university policies and guidelines mentioned above are specific to URMC, other institutions will have designed and implemented their own unique policies and guidelines related to bias and discrimination. Facilitators who choose to introduce this intervention at other institutions should reference their own institutional policies and guidelines. It is critical to link these educational sessions to institutional policies and guidelines that are in place to protect health care professionals from bias and discrimination; it is also important to observe federal antidiscrimination laws passed in 1964. That being said, appropriate laws, policies, and guidelines are necessary but insufficient to change culture.

Aristotle's general moral theory provided the foundation for the remainder of each session.[9] This theory supported the ethical framework of the personal, the professional, and the practice used to teach bioethics at URSMD and URMC. Ethical action begins with the personal; individuals must acknowledge their own beliefs, values, and reactions to other individuals and situations they encounter. This requires reflection on the influences on one's life, including the structural racism inherent in society in the United States. The professional requires individuals to consider their professional responsibilities in the context of their personal values. The practice requires individuals to apply their acknowledgment of personal and professional factors when making clinical decisions.

The grand rounds and invited workshops in the IM and EM departments then called for audience participation in the reenactment of a prefilmed scenario (Appendix C), in which a health care team was confronted with a patient expressing explicit religious bias. This prefilmed scenario was based on an actual experience by a URMC health care professional. The then-director of the standardized patient program and two medical students volunteered to participate in the filming. Of note, the individual playing the antagonist requested that participants

Copyright © 2020 Rizk et al. This is an open-access publication distributed under the terms of the Creative Commons Attribution license.

in the program be reminded they were playing a role and to please not ascribe the values of the character they played to them; at all stages, this work took courage. The purpose of using a prefilmed scenario in these sessions was to utilize the limited time efficiently while still providing audience members with direct exposure to the modified version of FT.

In addition, audience members were given an opportunity to playact the scene, and spect-actors were asked to generate and demonstrate alternative solutions to the problem of oppression. A summary of the prefilmed scenario is outlined below:

> Ms. Brown is a 50-year-old white female with an exacerbation of chronic lung disease admitted to the hospital. Although she has a cough and is mildly short of breath, she is otherwise medically stable. Two residents enter Ms. Brown's room: Dr. Khan, a second-year female resident who is Muslim and wears a hijab, and Dr. Jensen, a first-year male resident who appears white. Dr. Khan extends her hand to greet Ms. Brown. Refusing to shake her hand, Ms. Brown folds her arms and looks away. Dr. Khan introduces herself and informs Ms. Brown that she and Dr. Jensen will be caring for her while she is in the hospital. Ms. Brown eventually states that she does not want a Muslim woman for a doctor. She insists that Dr. Jensen be her doctor and demands that Dr. Khan leave the room. Dr. Jensen remains silent.

The invited series for the Department of Psychiatry presented FT in a way distinct from the two previous categories of sessions (Table 2). Offering multiple sessions over the course of the summer allowed for an expansion of the materials used by facilitators. In the invited series, participants engaged in a live demonstration of the work presented in Beyond Observation: Developing Clinical Competencies at the Art Gallery,[26] played games,[27] and, rather than using a prefilmed scenario, were taught how to write and direct scenarios based on their own personal encounters with bias in the workplace. Each original scenario was required to include at least three character types each with a unique role within the cycle of oppression—antagonist, protagonist, and bystander. Participants selected several scripts to develop further during the course of the workshop.

At the end of each session, participants were asked to complete an evaluation form. The grand rounds evaluation used a satisfaction-type questionnaire, the invited workshops used an "I learned"–type questionnaire, and the invited series used a combined satisfaction-type and clinical competency–type questionnaire. These evaluation forms differed from one another based on the format of the sessions. All evaluation forms were voluntary and anonymous and were reviewed to ensure that participant feedback was incorporated into future sessions. Once all sessions had been completed, the facilitators created a standard evaluation form incorporating what were believed to be the most comprehensive yet relevant categories of feedback. We recommend the use of this standard evaluation form following all further educational sessions (Appendix D).

Table 2. Breakdown of the Invited Series in the Department of Psychiatry at University of Rochester Medical Center

Session	Time	Title	Description
1	50 min.	Introduction to Theater of the Oppressed: The Methods and Seeing Differently	This session engaged participants in a live demonstration of the work presented in Beyond Observation: Developing Clinical Competencies at the Art Gallery.
2	50 min.	Connecting the Five Senses and Playing Games	This session introduced participants to games from Augusto Boal's *Games for Actors and Non-Actors* (Routledge; 1992).
3	50 min.	Identification of Department Culture	This session taught participants the formula for developing a script to use in forum theater and facilitated small groups in creation of scripts from actual incidents or events.
4	50 min.	Forum Theater Activities	Participants presented a scene and used forum theater to create alternate outcomes.
5	50 min.	Forum Theater Activities	Participants presented a scene and used forum theater to create alternate outcomes.
6	50 min.	Forum Theater Activities	Participants presented a scene and used forum theater to create alternate outcomes.
7	50 min.	Forum Theater Debrief	This session provided an opportunity for participants to discuss the learning activities from the previous sessions, offer feedback about sessions, and express ideas for future programs.

Results

For the grand rounds surveys, 58 out of 127 participants responded. These evaluations were based on the standard forms for continuing medical education used at the institution. Approximately seven physicians, 15 social workers, 16 nurses, 12 staff, and seven assorted others were represented in the survey respondents. For the invited workshop surveys, 38 out of 62 participants responded. These evaluations differed from the ones used in the grand rounds, as the facilitators and the residency directors modified the questions to more directly reflect the updated format and the target audience. Survey

Copyright © 2020 Rizk et al. This is an open-access publication distributed under the terms of the Creative Commons Attribution license.

respondents included both residents and fellows from the respective departments. For the invited series surveys, 61 out of an unknown total number of participants responded over the course of seven sessions. These evaluations differed from the ones used in the grand rounds and invited workshops, again because the facilitators modified the questions to more specifically reflect the updated format and the target audience. Fourteen physicians, 17 social workers, five PhDs, and 25 assorted others were represented in the survey respondents. One limitation of the invited series surveys was that while some learners attended one workshop and some learners attended multiple workshops, no records were kept to determine how many workshops each learner attended.

Participants in all sessions provided feedback on both the content and the pedagogy of the FT tool. The following section reports themes from the iterative process and includes data from all sessions.

Summary of Feedback on Content of the FT Tool

The grand rounds were held with more than a hundred total participants comprising members of the community at URSMD and URMC. We asked grand rounds participants to rate parameters related to content: relevance and accuracy, as well as interest and value. Both questions elicited largely positive responses, with both receiving a significant majority of *excellent* responses (86%). (There were no ratings lower than *average* on the scale for any of the parameters being assessed in the survey.) The primary theme that arose from the open-ended comments for this session was the importance of the topic of expressed racial bias and discrimination.

The invited workshops were held with 20 IM residents and fellows and 18 EM residents and fellows. We asked participants to rate four statements related to session content. Two of those statements are relevant here: “I learned how to respond to patients who express bias” and “I learned how to demonstrate support to a colleague who is the target of expressed bias.” Regarding the first statement, 65% of IM participants and 17% of EM participants strongly agreed that they had learned how to respond to patients who expressed bias (Figure 1). Regarding the second statement, 75% of IM participants and 33% of EM participants strongly agreed that they had learned how to demonstrate support to a colleague who was the target of expressed bias (Figure 2). The primary theme that arose from the open-ended comments for these sessions was the need for more time to explore the relevant institutional policies and guidelines and how to implement them in a workplace setting directly applicable to the participants. We predict that this desire to rework the session to make it more relevant to the target audience explains the difference in responses between the IM residents and the EM residents; the EM residents pointed out that their workplace environment was distinct in its quick pace and frontline culture.

The invited series was held with an unknown number of participants comprising interdisciplinary teams of health care professionals in the Department of Psychiatry. We asked

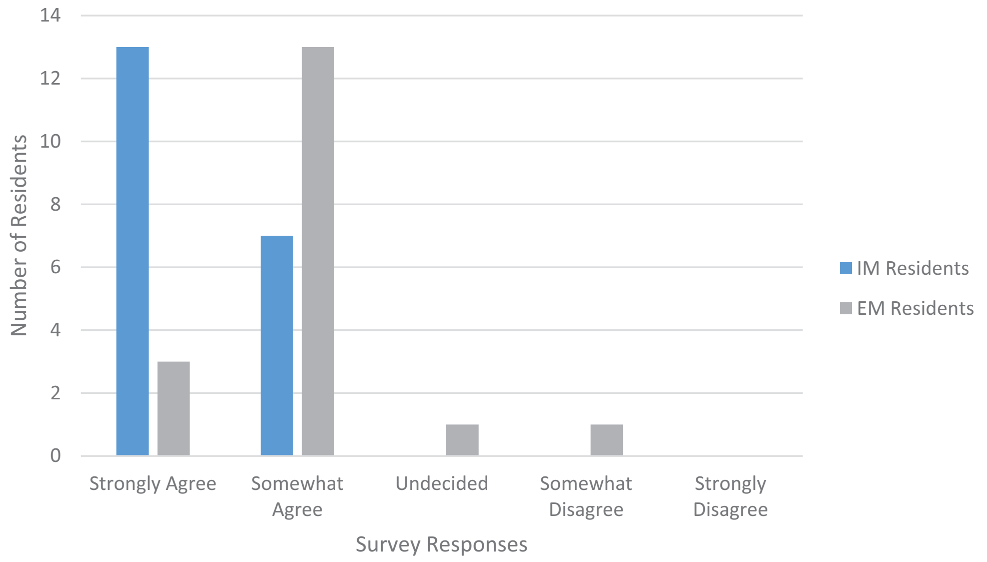

Figure 1. Participant responses to the statement “I learned how to respond to patients who express bias” following the invited workshop sessions. Abbreviations: EM, emergency medicine; IM, internal medicine.

Copyright © 2020 Rizk et al. This is an open-access publication distributed under the terms of the Creative Commons Attribution license.

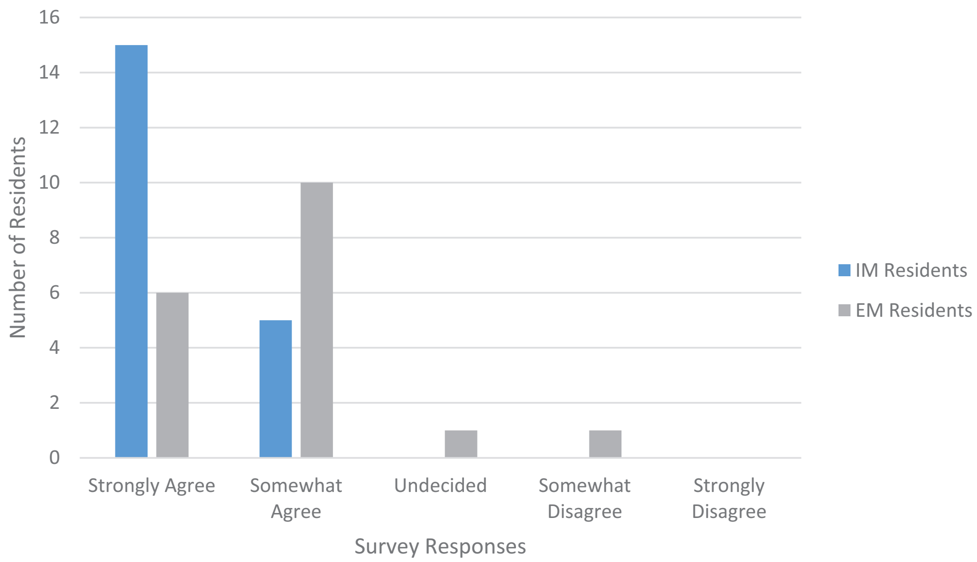

Figure 2. Participant responses to the statement “I learned how to demonstrate support to a colleague who is the target of expressed bias” following the invited workshop sessions. Abbreviations: EM, emergency medicine; IM, internal medicine.

participants to rate a series of categories related to session content on a 5-point scale. One of those categories is relevant here: “Please rate the impact today’s presentation will have on your practice of these core competencies for clinical practice,” with a further breakdown into subcategories of specific core competencies. Across sessions, responses of 5 out of 5 points varied from approximately 50% to 80% (a reminder to the reader that numerical values are not provided because the invited series questionnaires did not track duplicate participants in the workshops). The primary theme that arose from the open-ended comments for these sessions was an appreciation for the opportunity to engage in a challenging yet meaningful intervention to explore the complicated and pervasive topic of racial bias and discrimination through theater.

Summary of Feedback on the Pedagogy of the FT Tool

For each session, we also included questions or invited comments about the logistics of the pedagogy of FT. Grand rounds participants provided *excellent* responses for the quality of the presentation (72%), the pace of the presentation (77%), and the sequence of the presentation (70%). This session received a number of comments suggesting that the intervention be expanded into a workshop or a series to provide participants with more time for an exploration of the themes of racial bias in the health care workplace.

Invited series participants filled out an evaluation form similar to the one used in the grand rounds; these participants also largely provided *excellent* responses for the quality of the presentation, the pace of the presentation, and the sequence of the presentation (a reminder to the reader that numerical values are not provided because the invited series questionnaires did not track duplicate participants in the workshops). However, this session also received a number of comments, this time expressing an appreciation for the small-group setting of the series, although also recommending redesigning the series to include sessions of longer duration.

Discussion

Bias and discrimination targeting health care professionals from underrepresented and racial minorities have been identified as a powerful force within the health care workplace. However, there is limited, if any, opportunity for individuals to learn about institutional antidiscrimination policies or to practice strategies for responding to patient expressions of racial bias. Our intervention used FT to provide health care professionals with the tools needed to identify and expose racial bias without causing harm to the therapeutic relationship and with practice implementing these tools in a supportive and structured environment. The form of these sessions evolved over time, becoming more comprehensive and more involved with each subsequent iteration.

The goal of the initial grand rounds was to raise awareness of implicit and explicit bias in clinical care. By focusing on bias and discrimination exhibited by patients, the facilitators intentionally structured the intervention so that participants were all on the same team. The sessions were designed to create a space

Copyright © 2020 Rizk et al. This is an open-access publication distributed under the terms of the Creative Commons Attribution license.

that fostered a greater appreciation of the impact of bias and discrimination and, by focusing on the biases of others, tended to discourage participants' defensiveness. This strategy worked. Participants expressed outrage at the content of the video, especially those who rarely, if ever, faced such expressions of hostility directed at their identity. This reaction to the video provided motivation to actively participate in the challenging work. One of the challenges of this work is that participants are asked to volunteer and expose themselves in front of their colleagues in an experiential situation that is particularly distressing. Many are unprepared to do this work because racial bias may not be part of their lived experience.

Importantly, this pilot program began as a teaching initiative rather than a research project. Therefore, certain limits applied—the sessions were organically created, the process was nonlinear, the evaluation tools were inconsistent, the data collected for the multiple sessions did not include reduplication, and the percentages were not analyzed for statistical significance but rather to indicate rough proportions. In addition, each of the three forms of instruction used different evaluation tools that were designed to reflect the target audience and the stated goals. Even so, we were able to confirm that our learning objectives were met by conducting a qualitative analysis of the feedback provided by participants in each session—in the form of both verbal comments and written comments.

Although a variety of protections have been put in place to address issues of patient bias and discrimination towards health care professionals, a number of questions remain: How exactly should they be applied to clinical practice? What do professionals do when they realize they are in the presence of a patient who is expressing bias? Through the discussion and playacting facilitated by our sessions, health care professionals were challenged to answer those questions through dialogue and participation in realistic scenarios during which they examined different methods of addressing expressed bias.

A critical feature of FT is that participants create solutions they can employ in practice. Upon demonstration of a successful approach, the facilitators would ask the participants/spect-actors if they could utilize the same approach. On occasion, a participant would respond that as much as they appreciated their colleagues' approach, they could not replicate it. The group would then replay the scenario, allowing that participant the opportunity to adjust the performance to be one they could comfortably offer. Every person needs to develop the capacity to appropriately respond to inappropriate and morally challenging behaviors. This does not require the same kind of response from every person, as the same message can be delivered in multiple ways. Having all team members collaboratively develop this capacity demonstrates a common value and strengthens team relationships.

Overall, the highly novel approach of using FT as a teaching tool for how to manage bias and discrimination by patients and their families was received very enthusiastically by a wide array of learners, including physicians, nurses, social workers, and others. Participants reported significant improvements in learning how to manage bias and discrimination by patients, as both targets of oppression and witnesses to oppression. Lessons learned related to content, pedagogy, and design planning. Regarding content, we learned that the FT model needs to be carefully tailored for different types of learners. Regarding pedagogy, we heard from all types of learners that there is a need for longer and more frequent sessions in order to better achieve the stated learning objectives.

We strongly encourage other health care institutions across the country to recreate this pilot project and offer this educational activity. We suggest that self-identified facilitators take time to research the relevant policies and guidelines at their home institutions and review the material we provide in order to effectively introduce this method of education. First, we recommend offering an initial session in the form of a grand rounds. This exposes institutional leaders and administrators to the concept of FT, gauges participants' understanding of their institutional culture, and hopefully leads to an invitation for more in-depth sessions with individuals' groups or departments. This invitation is critical for three reasons: (1) It indicates that the particular group or department has identified a need for a culture change, (2) it demonstrates the desire of the particular group or department to actively engage in strategies that facilitate culture change, and (3) it avoids the risk of facilitators imposing a predetermined agenda onto unwilling or unready participants.

Furthermore, we believe that this pilot demonstrates the worthiness of future empirical research to evaluate the model of FT. Research outcomes should include factors proven to change clinical practice, such as provider levels of comfort with managing patients who exhibit bias. We recommend using the standard evaluation form (Appendix D), which has questions measuring self-reported opinions about the impact the presentation will have on the practice of core competencies for clinical practice. Such changes may lead to improving the competence and comfort of health care professionals when dealing with bias and discrimination expressed by patients and their families.

Copyright © 2020 Rizk et al. This is an open-access publication distributed under the terms of the Creative Commons Attribution license.

Appendices

A. Presentation.pptx

B. Facilitator Guide.docx

C. Prefilmed Scenario.m4v

D. Evaluation Form.docx

All appendices are peer reviewed as integral parts of the Original Publication.

Nina Rizk, MPH, MS: Medical Student, University of Rochester School of Medicine and Dentistry

Shaunpaul Jones: Medical Student, University of Rochester School of Medicine and Dentistry

Margie Hodges Shaw, JD, PhD, HEC-C: Associate Professor, Department of Medical Humanities and Bioethics, University of Rochester School of Medicine and Dentistry; Director of the Law and Bioethics Theme, University of Rochester School of Medicine and Dentistry; ORCID: https://orcid.org/0000-0002-9068-9029

Adrienne Morgan, PhD: Associate Vice President of Equity and Inclusion, University of Rochester School of Medicine and Dentistry; Assistant Professor, Department of Medical Humanities and Bioethics, University of Rochester School of Medicine and Dentistry

Acknowledgments
The authors would like to thank Katherine Burke, MFA, project coordinator, The Art and Practice of Medicine, Lerner College of Medicine (https://katherinemburke.wordpress.com/about/), and Carli Gaughf, MA, founder of Theatre for Healthcare Equity, for providing training on the theater of the oppressed and forum theater (https://www.carligaughf.com/). The authors also thank Molly McNulty, JD, for editing support.

Disclosures
None to report.

Funding/Support
The Paul M. Schyve, MD Center for Bioethics at the University of Rochester awarded Nina Rizk and Shaunpaul Jones the 2018 Summer Medical School Fellowship in support of this project. The Institute for Innovative Education provided funding to bring Ms. Katherine Burke to Rochester and to send the facilitators to the workshop in South Carolina. The University of Rochester School of Medicine and Dentistry and the University of Rochester Medical Center provided funding to bring Ms. Carli Gaughf to Rochester.

Ethical Approval
The University of Rochester Office of Human Subjects Protection approved this study.

References

1. Watson S. Credentials don't shield doctors, nurses from bias. WebMD. October 18, 2017. https://www.webmd.com/a-to-z-guides/news/20171018/survey-patient-bias-toward-doctors-nurses
2. Crutcher RA, Szafran O, Woloschuk W, Chatur F, Hansen C. Family medicine graduates' perceptions of intimidation, harassment, and discrimination during residency training. *BMC Med Educ*. 2011;11:88. https://doi.org/10.1186/1472-6920-11-88
3. March C, Walker LW, Toto RL, Choi S, Reis EC, Dewar S. Experiential communications curriculum to improve resident preparedness when responding to discriminatory comments in the workplace. *J Grad Med Educ*. 2018;10(3):306-310. https://doi.org/10.4300/JGME-D-17-00913.1
4. Hu YY, Ellis RJ, Hewitt DB, et al. Discrimination, abuse, harassment, and burnout in surgical residency training. *N Engl J Med*. 2019;381(18):1741-1752. https://doi.org/10.1056/NEJMsa1903759
5. Weeks LD. When the patient is racist, how should the doctor respond? STAT. June 12, 2017. https://www.statnews.com/2017/06/12/racism-bias-patients-doctors/
6. Okwerekwu JA. The patient called me a "colored girl." The senior doctor training me said nothing. STAT. April 11, 2016. https://www.statnews.com/2016/04/11/racism-medical-education/
7. Jain SH. The racist patient. *Ann Intern Med*. 2013;158(8):632. https://doi.org/10.7326/0003-4819-158-8-201304160-00010
8. Paul-Emile K, Smith AK, Lo B, Fernández A. Dealing with racist patients. *N Engl J Med*. 2016;374(8):708-711. https://doi.org/10.1056/NEJMp1514939
9. Shaw MH, D'Angio CT, Dadiz R. Educational perspectives: personal, professional, and practice—a framework for ethics education. *Neoreviews*. 2016;17(2):e61-e69. https://doi.org/10.1542/neo.17-2-e61
10. Whitgob EE, Blankenburg RL, Bogetz AL. The discriminatory patient and family: strategies to address discrimination towards trainees. *Acad Med*. 2016;91(11):S64-S69. https://doi.org/10.1097/ACM.0000000000001357
11. Wilkins KM, Goldenberg MN, Cyrus KD. ERASE-ing patient mistreatment of trainees: faculty workshop. *MedEdPORTAL*. 2019;15:10865. https://doi.org/10.15766/mep_2374-8265.10865
12. Eisenberg A, Rosenthal S, Schlussel YR. Medicine as a performing art: what we can learn about empathic communication from theater arts. *Acad Med*. 2015;90(3):272-276. https://doi.org/10.1097/ACM.0000000000000626
13. Grieco M, Chambliss C. Educational methods for addressing diversity issues: the use of sociodramatic techniques. ResearchGate. January 2001. https://www.researchgate.net/

Copyright © 2020 Rizk et al. This is an open-access publication distributed under the terms of the Creative Commons Attribution license.

publication/234560224_Educational_Methods_for_Addressing_Diversity_Issues_The_Use_of_Sociodramatic_Techniques

14. Watson K. Perspective: serious play: teaching medical skills with improvisational theater techniques. *Acad Med*. 2011;86(10): 1260-1265. https://doi.org/10.1097/ACM.0b013e31822cf858
15. Langlois S, Teicher J, Derochie A, Jethava V, Molley S, Nauth S. Understanding partnerships with patients/clients in a team context through verbatim theater. *MedEdPORTAL*. 2017; 13:10625. https://doi.org/10.15766/mep_2374-8265.10625
16. Wolfe AD, Hoang KB, Denniston SF. Teaching conflict resolution in medicine: lessons from business, diplomacy, and theatre. *MedEdPORTAL*. 2018;14:10672. https://doi.org/10.15766/mep_2374-8265.10672
17. Hoffmann-Longtin K, Rossing JP, Weinstein E. Twelve tips for using applied improvisation in medical education. *Med Teach*. 2018;40(4):351-356. https://doi.org/10.1080/0142159X.2017.1387239
18. Nagji A, Brett-MacLean P, Breault L. Exploring the benefits of an optional theatre module on medical student well-being. *Teach Learn Med*. 2013;25(3):201-206. https://doi.org/10.1080/10401334.2013.801774
19. Bell SK, Pascucci R, Fancy K, Coleman K, Zurakowski D, Meyer EC. The educational value of improvisational actors to teach communication and relational skills: perspectives of interprofessional learners, faculty, and actors. *Patient Educ Couns*. 2014;96(3):381-388. https://doi.org/10.1016/j.pec.2014.07.001
20. Hobson WL, Hoffmann-Longtin K, Loue S, et al. Active learning on center stage: theater as a tool for medical education. *MedEdPORTAL*. 2019;15:10801. https://doi.org/10.15766/mep_2374-8265.10801
21. Freire P. *Pedagogy of the Oppressed*. Herder and Herder; 1970.
22. Boal A. *Theatre of the Oppressed*. Theatre Communications Group; 1985.
23. Bleakley A. *Medical Humanities and Medical Education: How the Medical Humanities Can Shape Better Doctors*. Routledge; 2015.
24. University of Rochester. University policy/procedure: policy 106: policy against discrimination, harassment, and discriminatory employment/service practices. University of Rochester Office of Human Resources. Updated September 15, 2020. https://www.rochester.edu/working/hr/policies/pdfpolicies/106.pdf
25. University of Rochester–Strong Memorial Hospital. Policy/procedure: policy 4189811: assuring a respectful and inclusive environment. Revised November 11, 2017. https://urmc-smh.policystat.com/policy/4189811/
26. Clark SB, Daiss SDP. Beyond observation: developing clinical competencies at the art gallery. University of Rochester Medical Center: Five Question Protocol. 2017-2018. https://medhum.digitalscholar.rochester.edu/
27. Boal A. *Games for Actors and Non-Actors*. Routledge; 1992.

Received: March 9, 2020
Accepted: July 15, 2020
Published: November 20, 2020

Copyright © 2020 Rizk et al. This is an open-access publication distributed under the terms of the Creative Commons Attribution license.
